# Discovery of a pyrrole-pyridinimidazole derivative as novel SIRT6 inhibitor for sensitizing pancreatic cancer to gemcitabine

**DOI:** 10.1038/s41419-023-06018-1

**Published:** 2023-08-04

**Authors:** Nannan Song, Xian Guan, Siqi Zhang, Yanqing Wang, Xuekai Wang, Zhongxia Lu, Daochen Chong, Jennifer Yiyang Wang, Rilei Yu, Wengong Yu, Tao Jiang, Yuchao Gu

**Affiliations:** 1 Key Laboratory of Marine Drugs, Ministry of Education, School of Medicine and Pharmacy, Ocean University of China, Qingdao, 266003 China; 2 Department of Pathology, 971 Hospital of PLA Navy, Qingdao, 266071 China; 3 Laboratory for Marine Drugs and Bioproducts of Qingdao National Laboratory for Marine Science and Technology, Qingdao, 266237 China

**Keywords:** Virtual screening, Pancreatic cancer

## Abstract

Pancreatic cancer is a highly aggressive cancer, and is primarily treated with gemcitabine, with increasing resistance. SIRT6 as a member of sirtuin family plays important roles in lifespan and diverse diseases, such as cancer, diabetes, inflammation and neurodegenerative diseases. Considering the role of SIRT6 in the cytoprotective effect, it might be a potential anticancer drug target, and is associated with resistance to anticancer therapy. However, very few SIRT6 inhibitors have been reported. Here, we reported the discovery of a pyrrole-pyridinimidazole derivative, 8a, as a new non-competitive SIRT6 inhibitor, and studied its roles and mechanisms in the antitumor activity and sensitization of pancreatic cancer to gemcitabine. Firstly, we found a potent SIRT6 inhibitor compound 8a by virtual screening and identified by molecular and cellular SIRT6 activity assays. 8a could effectively inhibit SIRT6 deacetylation activity with IC_50_ values of 7.46 ± 0.79 μM in FLUOR DE LYS assay, and 8a significantly increased the acetylation levels of H3 in cells. Then, we found that 8a could inhibit the cell proliferation and induce cell apoptosis in pancreatic cancer cells. We further demonstrate that 8a sensitize pancreatic cancer cells to gemcitabine via reversing the activation of PI3K/AKT/mTOR and ERK signaling pathways induced by gemcitabine and blocking the DNA damage repair pathway. Moreover, combination of 8a and gemcitabine induces cooperative antitumor activity in pancreatic cancer xenograft model in vivo. Overall, we demonstrate that 8a, a novel SIRT6 inhibitor, could be a promising potential drug candidate for pancreatic cancer treatment.

## Introduction

Pancreatic cancer is a highly aggressive cancer with a 5-year survival rate of 11% [1]. Gemcitabine is still the first-line chemotherapy for pancreatic cancer [2]. However, high rate of chemoresistance severely impedes its efficacy as a cornerstone of pancreatic cancer chemotherapy [3]. Therefore, there is an urgent need for developing innovative targets and novel therapeutic strategies for therapeutic intervention of pancreatic cancer.

SIRT6 as a member of sirtuin family plays important roles in gene transcription, DNA repair, metabolic and glucose homeostasis [4–7]. SIRT6-deficient mice have displayed genomic instability, premature senility and finally dying at ~4 weeks [8]. SIRT6 knockout cells fail to repair multiple types of DNA damage, including DNA double-strand break repair and base excision repair responding to genotoxic and oxidative stress [8–10]. There are many factors leading to resistance to chemotherapeutic agents of cancer, including activation of DNA damage repair pathway, the stemness of cancer cells and immune evasion [11]. Based on the role of DNA damage repair in chemotherapeutic drug resistance, several inhibitors targeting DNA repair mechanisms, such as the single strand break repair protein PARP inhibitors, are being studied in clinical trials for the treatment of cancer [12, 13]. Due to the role of SIRT6 in the regulation of DNA repair, mounting evidence points towards SIRT6 may be a target of tumor chemotherapy drug resistance [14]. Therefore, SIRT6 inhibitors might be helpful to improve the efficacy of currently available therapeutics. However, very few SIRT6 inhibitors have been reported and have no ideal inhibitory efficacy [15, 16], and the antitumor efficacy of existing SIRT6 inhibitors is still weak and the underlying mechanism remains largely unknown [17, 18]. Hence, developing highly effective SIRT6 inhibitors is important in improving the therapeutic effect of pancreatic cancer.

Marine environment has demonstrated to be a prolific source of compounds with unique chemical features and structural activity due to the outstanding biological diversity [19, 20]. Marine natural products are ideal candidates as hit or lead compounds in new drug discovery and further development [21–23]. For the development and application of marine drugs, our group has established a fine three-dimensional structure database (http://mc3d.qnlm.ac/) containing 30,117 marine natural products by Ocean University of China that can be directly used for virtual drug screening and intelligent drug design. On this basis, we can quickly and efficiently discover targeted drugs for various diseases.

In this study, to discover novel SIRT6 inhibitors, we initially performed a virtual screening study against the marine natural products and derivatives library using the protein crystal of SIRT6. Verified by biological activity test, we found that compound 8a showed more potent inhibitory effect on the SIRT6 at cellular level. We further investigated the roles and mechanisms of compound 8a in antitumor activity and chemosensitivity in pancreatic cancer cells and xenograft models. This study showed that compound 8a is an effective SIRT6 inhibitor, and a potential drug candidate for pancreatic cancer treatment.

## Materials and methods

### Chemistry

All commercially available starting materials and solvents were purchased from commercial vendors and used without further purification. Reactions were monitored using analytical thin-layer chromatography on precoated silica gel GF254 plates (Qingdao Haiyang Chemical Plant, Qingdao, China) and visualized under ultraviolet light (254 nm and 365 nm). Column chromatography was performed on silica gel (200–300 mesh). ^1^H and ^13^C NMR spectra were recorded on the Broker AVANCE NEO and Agilent DD2 500 with 400 or 500 MHz for proton (^1^H NMR) and 100 or 125 MHz for carbon (^13^C NMR) with tetramethylsilane (Me_4_Si) as the internal standard, respectively. The chemical shifts (*δ*) were expressed in parts per million (ppm). Abbreviations used: s = singlet; d = doublet; t = triplet; q = quartet; m = multiplet. The coupling constant (*J*) values were described as hertz. High-resolution (ESI) MS spectra were recorded using a QTOF-2 Micromass spectrometer. The purity of the final compounds for biological evaluation was higher than 95% by analytical HPLC analysis with the Primaide 1210 system. General procedure to synthesis compounds (Supplementary information)

### Cloning, expression, and purification of recombinant human SIRT6

The human full-length SIRT6 gene was inserted into the prokaryotic expression vector (pET-28a (+)). The verified recombinant plasmid was transformed into Escherichia coli BL21 (DE3) expression strain and cultured overnight at 37 °C on LB plates supplemented with 50 μg/mL kanamycin. Single colonies were inoculated in fluid kanamycin-containing LB mediums. It was shaken at 160 rpm for 16 h at 37 °C and transferred to kanamycin-containing LB medium at a ratio of 1:100. It was shaken at 160 rpm at 37 °C until the OD_600_ reached 0.6. The Isopropyl-β-d-thiogalactopyranoside was added to make the final concentration of solution 0.5 mmol/L, cultured for 15 °C and 160 rpm for 24 h.

Bacterial liquid was harvested at 12,000 rpm at 4 °C for 20 min, and then re-suspended in Lysis buffer (20 mmol/L Hepes pH 7.5, 20 mmol/L imidazole, 5% Glycerol, 500 mmol/L NaCl). Cell pellets were disrupted by high-pressure crusher (JNBIO, Guangzhou China) and centrifuged (4 °C, 12000 rpm, for 20 min). Supernatant was collected and purified by FPLC with a 1 mL Ni–NTA Sepharose column (GE Healthcare, Stamford, USA). The SIRT6 protein was eluted at containing 50 mM, 100 mM, 150 mM, 200 mM, and 500 mM imidazole in elution buffer. The purified SIRT6 protein was dialyzed in dialysis buffer (50 mM Tris-HCl, 137 mM NaCl, 2.7 mM KCl, 1 mM MgCl_2_). The purified SIRT6 protein was analyzed by SDS-PAGE.

### In vitro SIRT6 deacetylation assays and IC_50_ determination

Fluor-De-Lys (FDL) assays were used for the examination of the deacetylation activities of sirtuins as described previously [24, 25]. For the detection of SIRT6 activity, the 50-μL reaction mixture contained 5 μM SIRT6, 2.5 mM NAD^+^ (β-Nicotinamide adenine dinucleotide hydrate, Sigma, N1636), 75 μM RHKK-Ac-AMC (GL Biochem, Shanghai), compounds/DMSO, and assay buffer. The reactions were incubated at 37 °C for 2 h, added 50 μL stop solution (40 mM nicotinamide, 6 mg/mL trypsin) at 25 °C for 30 min. Fluorescence intensity was measured at an excitation wavelength of 360 nm and an emission wavelength of 460 nm. IC_50_ values were calculated in GraphPad Prism version 8.00.

### Biolayer interferometry (BLI)

Binding assays were performed using Ni-NTA biosensors on the Octet® Red96 system (ForteBio, Menlo Park, CA) in 96-well microplates and measuring changes wavelength shift (in nm) over time (s). All steps were carried out at 25 °C with the plate shaking speed of 1000 rpm. The total working volume was 200 μL in 96-well microplate (Greiner Bio-one, Germany). The SIRT6 protein and compounds were diluted with phosphate-buffered saline (PBS, pH 7.4). Ni-NTA biosensors were prewetted for at least 10 min in PBS prior to the assay. Afterwards, the Ni-NTA biosensors were immobilized for 180 s with 200 μL culture containing 20 μg/mL SIRT6. After loading, sensors were washed in PBS buffer for another 120 s and transferred to wells containing different concentrations of compound 8a (0, 12.5, 25, 50, 100 μM) for binding. Finally, the dissociation step was carried out in kinetic buffer for 180 s. The assay data were processed and calculated using the ForteBio Octet RED analysis software.

### The cellular thermal shift assay (CETSA)

The CETSA [26] approach builds on the thermal shift assay concept where ligand binding to its target protein affects protein stability at high temperatures. Hela cells were plated in 100 mm plates. After 12 h attachment, Hela cells were incubated with 8a (25 μM) for 6 h, and then the cells were digested with trypsin and collected. The collected cells were subsequently aliquoted into six parts, and incubated each part at different temperatures (40, 41, 42, 43, 44, 45, 46, 47, 48, 49 and 50 °C) for 3 min. The heated cells were then snap frozen in liquid nitrogen three times to lyse. After that, the lysed cells were then centrifuged at 12,000 rpm for 20 min. Then the level of SIRT6 was detected by western blot.

### Cell culture

The BXPC-3, PANC-1, NIT-1, β-TC-6, and Hela cell lines were obtained from the Cell Resource Center of the Shanghai Institute for Biological Sciences, Chinese Academy of Sciences. All cell lines have been authenticated using STR profiling and tested for mycoplasma contamination. BXPC-3 were cultured in RPMI1640 medium (Gibco) supplemented with 10% fetal bovine serum, sodium pyruvate (1 mmol/L), penicillin (50 units/mL), and streptomycin (50 μg/mL). PANC-1 and Hela were maintained in Dulbecco’s modified Eagle’s medium (DMEM) supplemented with 10% (v/v) FBS, streptomycin (50 μg/mL) and penicillin (50 units/mL). NIT-1 were maintained in F-12K medium supplemented with 10% (v/v) FBS, streptomycin (50 μg/mL) and penicillin (50 units/mL). β-TC-6 were maintained in Dulbecco’s modified Eagle’s medium (DMEM) supplemented with 15% (v/v) FBS, streptomycin (50 μg/mL) and penicillin (50 units/mL). The cells were maintained in 37 °C incubator with 5% CO_2_.

### Animals

All animal experiments were performed in accordance with the principles and procedures approved by the Animal Ethics Committee of the Ocean University of China (Ethical Approval Numbers OUC-AE-2021-076). All animals were purchase from Beijing Vital River Laboratories (Beijing, China). All animals were allowed to adapt to the environment for 1 week upon arrival. These animals were kept in a standard environment with a 12-h light/dark cycle and allowing free drinking and eating. Mice were randomly allocated to experimental groups. No blinding method was used for injection. The sample sizes for the mice experiments were not predetermined using statistical methods and were based on the results of preliminary experiments. The sample size for each experiment is shown in the legend. No data were excluded from the analysis.

### Proliferation assay

BXPC-3 and PANC-1 cells were plated in 96-well plates at 5000 cells per well. After 12 h attachment, varying concentrations of 8a were added into the culture medium for 72 h. Then 10 μL MTT (5 mg/ml) solution was added into per well for 4 h at 37 °C. After removing medium, formazan crystals were dissolved in 150 µL DMSO. The cells viability was quantified by measuring the absorbance at 570 nm.

### Colony formation assay

The BXPC-3 cells were plated in six-well plates at 1000 cells per well. After 12 h, different concentrations of 8a, Gemcitabine or their combination were added for 7–10 days. The cells were fixed for 15 min with paraformaldehyde (4%) and then stained with crystal violet (0.1%) for 15 min at room temperature. Surviving colonies consisting of 50 or more cells were counted under microscope.

### Cell cycle analysis

The BXPC-3 cells were seeded onto six-well plates at 1 × 10^5^ cells per well and different concentrations of 8a, Gemcitabine or their combination were added for 48 h. The cells were washed with PBS, collected after trypsinization and fixed in cool 70% ethanol at −20 °C. After fixation, the cells were incubated in the dark with PBS containing 50 μg/mL propidium iodide and 100 μg/mL RNase A for 30 min at room temperature. Finally, the samples were analyzed on a flow cytometer (Beckman Coulter).

### Cell apoptosis analysis

To quantify cell apoptosis, BXPC-3 cells were treated with different concentrations of compound 8a, Gemcitabine or their combination for a further 72 h. Harvested cells were incubated with Muse™ Annexin V &Dead Cell assay kit (Muse TM Cell Analyzer, Millipore (catalog no. MCH100105)). Cells were then analyzed by flow cytometry (Muse TM Cell Analyzer, Millipore).

### Hoechst 33342 stain

BXPC-3 cells were seeded on the chamber-slides at 12-well plates and treated with the indicated concentration of compound 8a for 48 h. After treatment, cells were fixed with 4% paraformaldehyde for 15 min. Then, cells were stained with 10 μg/mL Hoechst 33342 for 30 min. After washing with PBS, slides were observed with fluorescent microscopy.

### SIRT6 siRNA transfection

The BXPC-3 cells were seeded onto 12-well plates. Human SIRT6 siRNA and negative control siRNA were transfected into BXPC-3 cells using Attractene Transfection Reagents (QIAGEN, Germany). After incubation for 72 h, the cells were harvested and lysed for protein analysis. The target sequence for si-SIRT6-1 is 5′- GGAAGAATGTGCCAAGTGT-3′, for si-SIRT6-2 is 5′- CAAGTTCGACACCACCTTT-3′, and for si-SIRT6-3 is 5′- GCTACGTTGACGAGGTCAT-3′.

### Cell glucose uptake assay

The NIT-1 and β-TC-6 cells were added to the wells of 12-well plates and incubated overnight. Then the culture medium without FBS was incubated for 6 h, and different concentrations of 8a (3.125, 6.25, 12.5 μM), insulin (100 nM) or their combination were added for 30 min. Glucose uptake was determined using a Glucose Uptake Assay Kit (Jiancheng, Nanjing).

### Glucose uptake and insulin secretion in vivo

Male C57BL/6 mice (18 ± 2 g, 5 weeks old) were randomly divided into four groups with six mice per group. Acute drug toxicity test was performed. Mice were fasted for 6 h, and basal blood glucose and serum insulin levels were measured by using Accu-Check Performa (Roche, Swiss) glucose meter and Mouse Insulin ELISA kit (Solarbio). Compound 8a (10, 20, 30 mg/kg) was intraperitoneally injected for 10 min, followed by glucose (2 g/kg) intraperitoneally injected. The blood glucose concentration was measured at 30 and 60 min after glucose injection, and the serum insulin content of mice was measured at 60 min. Compound 8a was treated every other day for 2 weeks and tested for chronic drug toxicity. According to the above method, the serum glucose and insulin content of mice were measured.

### Western blot analysis

Cells were harvested and lysed in a lysis buffer (solarbio, R0010) supplementing 1 mM PMSF and protease inhibitor (TargetMol, USA). Protein was separated on 7.5–15% SDS-PAGE gels and transferred to the 0.45 μm or 0.22 μm PVDF membrane (Merck Millipore), and subsequently blocked with 5% bovine serum albumin (BSA) in TBST. The blocked membrane was incubated with primary antibodies:SIRT6 (1:1000, Cell Signaling Technology, Cat#12486S, RRID:AB_2636969), Histone H3 Antibody (1:1000, Cell Signaling Technology, Cat#9715S, RRID:AB_331563), β-Actin (1:2000, Cell Signaling Technology, Cat#4970S, RRID:AB_2223172), Cleaved caspases-3 (1:1000, Cell Signaling Technology, Cat#9661S, RRID:AB_2341188), Cleaved Caspase-9 (Asp315) (1:1000, Cell Signaling Technology, Cat#20750S,RRID:AB_2798848), Cleaved PARP (1:1000, Cell Signaling Technology, Cat#5625S, RRID:AB_10699459), AKT (1:1000, Cell Signaling Technology, Cat#9272S, RRID:AB_329827), Phospho-AKT (Ser473) (D9E) (1:1000, Cell Signaling Technology, Cat#4060S, RRID:AB_2315049), ERK1/2 (1:1000, Cell Signaling Technology, Cat#4695S, RRID:AB_390779), Phospho-p44/42 MAPK (ERK1/2) (Thr202/Tyr204) (1:1000, Cell Signaling Technology, Cat#4370S, RRID:AB_2315112), Cyclin D1 (E3P5S) (1:1000, Cell Signaling Technology, Cat#55506 S, RRID:AB_2827374), Phospho-mTOR(Ser2448) (1:1000,Cell Signaling Technology, Cat#5536S, RRID:AB_10691552), Phospho-p70S6Kinase (Thr421/Ser424) (1:1000, Cell Signaling Technology, Cat#9204 S, RRID:AB_2265913), Phospho-Histone H2A.X (Ser139) (1:1000, Cell Signaling Technology, Cat#9718S, RRID:AB_2118009) at 4 °C overnight. After being washed with TBST, the membranes were incubated with horseradish peroxidase-conjugated secondary antibodies at room temperature for 1 h. Then, the membranes were detected by Tanon 5200 (Tanon, Beijing, China).

### Cells immunofluorescence assay

Cells were plated in the chamber-slides in the same conditions, and treated with compound 8a, gemcitabine or their combination for a further 48 h. Cells were washed twice with PBS and fixed with paraformaldehyde (4%) at room temperature for 15 min. After washing twice with PBS, cells were blocked in PBS containing 0.2% Triton X-100, 10% goat serum and 5% BSA for 2 h, and then incubated with primary antibodies (γ-H2AX, Ser139, Cell Signaling Technology, Cat#9718) overnight at 4 °C. Cells were stained with secondary antibodies (Alexa Fluor 488-conjugated anti-rabbit IgG, Abcam, Cat#ab150077) for 2 h at 4 °C in dark. After washing with PBS, the cells were stained with Hoechst and observed by confocal microscopy (Leica TCS SP8 STED 3X).

### Immunofluorescence of pancreatic tissues

Pancreatic tissue sections were incubated with primary antibodies overnight at 4 °C. The primary antibodies were as follows: anti-Glucagon (Cell Signaling Technology, Cat#2760), anti-insulin (Cell Signaling Technology, Cat#3014). The sections were stained with secondary antibodies (Alexa Fluor 488-conjugated anti-rabbit IgG, Abcam, Cat#ab150077) for 2 h at 4 °C in dark. After washing with PBS, the sections were stained with DAPI and observed by confocal microscopy (Leica TCS SP8 STED 3X).

### Tumor nude mice model

Female BALB/c nude mice (14 ± 2 g, 4 weeks old) were purchase. About 2 × 10^6^ of BXPC-3 cells were inoculated into nude mouse flanks. When the tumor volume reached approximately 100 mm^3^, mice were randomly divided into four groups with six mice per group before treatment and then treated with vehicle, 8a (20 mg/kg), gemcitabine (10 mg/kg) and their combination every 2 days. Tumor volumes and body weight were measured every 3 days, and use the formula to calculate the tumor volume: V = (L × W × W)/2 (V, tumor volume; L, length; and W, width). Mice were sacrificed and tumor tissues were removed. Tumor tissues were fixed with paraformaldehyde (4%) and embedded with paraffin.

### Statistical analysis

All the results were expressed as the mean ± SD. Each experiment was independently repeated three times in triplicate. Statistical analyses were performed with unpaired Student’s *t*-test or one-way ANOVA using GraphPad Prism version 8.0, and *p* < 0.05 was considered statistically significant.

## Results

### Discovery of hit compounds with the aid of molecular docking-based virtual screening and biological activity test

To obtain new inhibitors of SIRT6, we implemented virtual screening for marine natural compounds and their derivatives library on SIRT6 (PDB ID:5MF6) protein crystals. 16 compounds of Ageladine A derivatives were selected and chemically synthesized for biochemical assays. Then, we tested the inhibitory activity on each of these compounds at a fixed concentration of 25 μM by the FLUOR DE LYS (FDL) assay using a fluorogenic acetyl peptide substrate (RHKK-Ac-AMC). The chemical structures and bioactivities are given in Table 1. We found that compounds 5c, 6b, and 8a, containing a different isothiouronium-modified group, showed excellent inhibitory activity toward SIRT6. Compounds 5c and 6b, both of them with isothiourea displayed potent inhibition activity against SIRT6 at 25 μM with inhibition rate of 77.8% and 79.6%, respectively. Compound 6a with the length of four alkyl chains, did not show obvious activity against SIRT6 with inhibition rate of 26.7%. Compounds 8a, which contains tetramethyl-isothiourea, displayed modest inhibitory effect, with inhibition rate of 75.6%. These results indicated that by introducing isothiourea and tetramethyl-isothiourea to the pyrrole-pyridinimidazole through six alkyl chains, the compounds can greatly inhibit SIRT6. It also indicated that the different heteroring groups hardly contributed to the inhibition toward SIRT6. Therefore, we choose the three compounds (5c, 6b, and 8a) for follow-up biological evaluation.

**Table 1:** Inhibitory activity of Ageladine A derivatives towards SIRT6. The most significant inhibition is highlighted in red^a^.


Compounds	R_1_	*n*	R_2_	Salt form	Inhibition rate (%)	Scoring^b^
**1c**	CH_3_	/	H	/	3.7	−6.37
**2a**	H	/	H	/	4.1	−8.17
**2b**	H	/	H	CF_3_COOH	NI	−7.95
**4a**	H	6	Br	CF_3_COOH	5.3	−8.95
**5c**	CH_3_	6		/	77.8	−10.76
**6a**	H	4		HCl	26.7	−11.14
**6b**	H	6		HCl	79.6	−11.24
**8a**	H	6		HCl	75.6	−11.08
**10a**	H	6		CF_3_COOH	NI	−9.94
**10b**	H	6		CF_3_COOH	NI	−10.33
**9c**	SEM	6		/	NI	−7.65
**10c**	H	6		CF_3_COOH	6.4	−11.66
**10d**	H	6		CF_3_COOH	16.7	−6.54
**11a**	H	6	Br	/	NI	−8.23
**11b**		6		/	23.8	−10.67
**11c**		6		/	16.7	−8.80

^a^Each value of inhibition rate was calculated from 3 independent experiments performed in triplicate, NI = no inhibition.

^b^The SIRT6 protein (PDB ID:5MF6).

### Compound 8a inhibits SIRT6 deacetylation activity at cellular level

**Fig. 1:**
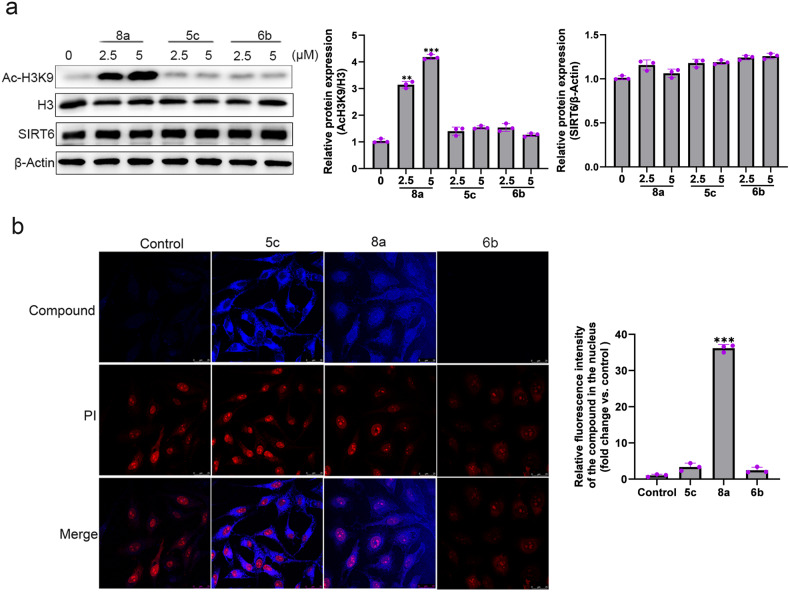
Effects of compounds 5c, 6b, and 8a on H3K9 acetylation in cells. **a** Hela cells were treated with compounds 5c, 6b, and 8a. The expression of acetylated H3K9, H3, and SIRT6 were analyzed by western blot. **b** Intracellular localization of these compounds. Nuclei shown in red are stained using PI, compounds in blue. Merged image shows the localization of compounds (63 × magnification, scale bar: 25 μm).

To investigate the SIRT6 activity at cellular levels, we examined the acetylation status of H3K9, which is a well-known SIRT6 substrate [27]. As shown in Fig. 1a, 8a significantly increased the acetylation levels of H3K9 in Hela cells in a dose-dependent manner, but 5c and 6b only slightly elevated the acetylation levels of H3K9, which were inconsistent with the in vitro assay. As these compounds can show violet fluorescence, intracellular localizations of these compounds were evaluated by confocal microscopy. We found that 8a was localized in the cytoplasm and nuclear, but 5c only accumulated in cell cytoplasm and 6b could not be absorbed into the cells (Fig. 1b). Since SIRT6 is predominantly located in the nucleus [28], we speculated that the most potent SIRT6 inhibitory activity of 8a at cellular levels can be attributed to its good cell penetration.

### Compound 8a is a non-competitive inhibitor of SIRT6

**Fig. 2:**
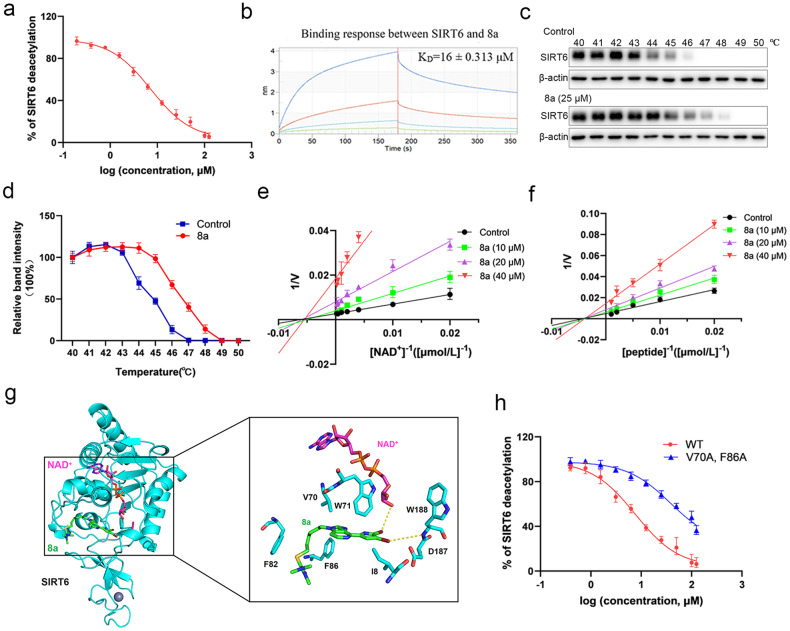
Compound 8a is a non-competitive inhibitor of SIRT6. **a** Dose-dependent deacetylation inhibition of SIRT6 by 8a, determined with RHKK-Ac-AMC by the FDL assay. **b** The binding effect of 8a on SIRT6 was analyzed by the Octet RED biolayer interferometry system. The real-time binding response (nm) was measured in seconds (s). **c**, **d** Hela cells incubated with or without 8a (25 μM) for 6 h were subjected to CETSA assay. SIRT6 was normalized with β-Actin. **e** Competition assay between NAD^+^ and 8a, as evaluated by the Fluor-De-Lys (FDL) assay. **f** Competition assay between RHKK-Ac-AMC and 8a, as evaluated by the Fluor-De-Lys (FDL) assay. **g** Details of the interaction of SIRT6 with 8a. SIRT6 is represented as cyan cartoon, 8a, NAD^+^ and key residues are represented by green, magenta and gray sticks, respectively. The yellow dashed lines indicate hydrogen bonds. **h** Effect of 8a on deacetylation activity of SIRT6 mutants, as determined by FDL assays. Data are presented as the mean ± SD, *n* = 3 wells, from three independent experiments.

Based on the above results, 8a was further investigated in this study. We found that 8a could effectively inhibit SIRT6 with IC_50_ values of 7.46 ± 0.79 μM by FDL assay (Fig. 2a). To further evaluate the selectivity of 8a for SIRT6, we examined the effect of 8a on the deacetylation activity of other HDAC family members, including HDAC1-HDAC11, SIRT1-SIRT3 and SIRT5. As shown in Table S1, 8a showed no activity for SIRT3, SIRT5, HDAC1, HDAC2, HDAC4, HDAC5, HDAC7, and HDAC9-HDAC11 at concentrations up to 200 μM. The IC_50_ of 8a on SIRT1, SIRT2, HDAC3, HDAC6, and HDAC8 were 80.52 ± 1.91 μM, 92.21 ± 1.95 μM, 111.9 ± 2.05 μM, 96.77 ± 1.98 μM, and 102 ± 2.01 μM, respectively, much higher than the IC_50_ of 8a on SIRT6 (7.46 ± 0.79 μM). Therefore, these results suggest that 8a was a selective SIRT6 inhibitor. To examine the binding affinity of 8a and SIRT6, BLI technology was used and the results showed a high affinity value (KD = 16 ± 0.313 μM) (Fig. 2b). Furthermore, to confirm binding of 8a to SIRT6 in cells, we carried out the cellular thermal shift assay (CETSA). The CETSA approach builds on the thermal shift assay concept where ligand binding to its target protein enhances the target protein stability. We observed that the thermal stability of SIRT6 increased after 8a treatment (Figs. 2c, d). Altogether, the above results indicated that 8a can directly bind to SIRT6 and inhibit its deacetylation activity.

To investigate whether compound 8a is a competitive inhibitor, we performed competition assays between the substrates and compound 8a. As shown in Fig. S1, increasing concentrations of the substrate peptide RHKK-Ac-AMC or NAD^+^ had no effect on the inhibitory activity of 8a. Then, we carried out an enzymatic kinetic assay to further clarify the inhibition mode of 8a (Fig. 2e, f). Lineweaver–Burk plot analysis revealed that 8a did not alter the K_m_ but obviously decreased V_max_ for both the RHKK-Ac-AMC and NAD^+^ substrates. These findings demonstrated that 8a is a non-competitive inhibitor.

To explore the interaction mechanism, we performed the molecular docking experiment between SIRT6 (PDB ID: 6QCJ) and 8a. The results showed that the binding energy between 8a and SIRT6 was −6.9 kcal/mol, with a strong binding affinity between them. 8a binds in the catalytic channel of SIRT6 protein, but it does not occupy the binding site of coenzyme NAD^+^ or substrate peptide. 8a mainly formed hydrogen bond with the backbone amino group of D187 and the ribose hydroxyl group of the coenzyme NAD^+^, and stabilized the whole system by hydrophobic interactions with key amino acids such as I8, V70, W71, F82 and F86 (Fig. 2g). To verify this binding model, the mutant (V70A and F86A) of SIRT6 was constructed by site mutation. The mutation did not affect the deacetylation activity of SIRT6, but the IC_50_ value of 8a to the mutant increased approximately 10-fold (IC_50_ = 79 ± 9 μM) (Fig. 2h). Moreover, there were also some other potential binding pockets predicted by molecular docking (data not shown). Based on the prediction, we constructed other two SIRT6 mutants (E140A/R195A and T184A/S191A/P193A/E283A). Additionally, two amino acid residues in the catalytic channel of SIRT6 protein, which are non-critical for the NAD^+^ binding, were mutated (E22A and D63A). All the three mutants did not significantly affect the inhibitory activity of 8a (Fig. S2). Collectively, 8a, as a non-competitive inhibitor of SIRT6 protein, achieved the effect of inhibiting the deacetylation activity of SIRT6 protein mainly by occupying the key catalytic region of SIRT6 protein.

### Compound 8a inhibits cell proliferation, induces cell-cycle arrest and apoptosis in PDAC cells

**Fig. 3:**
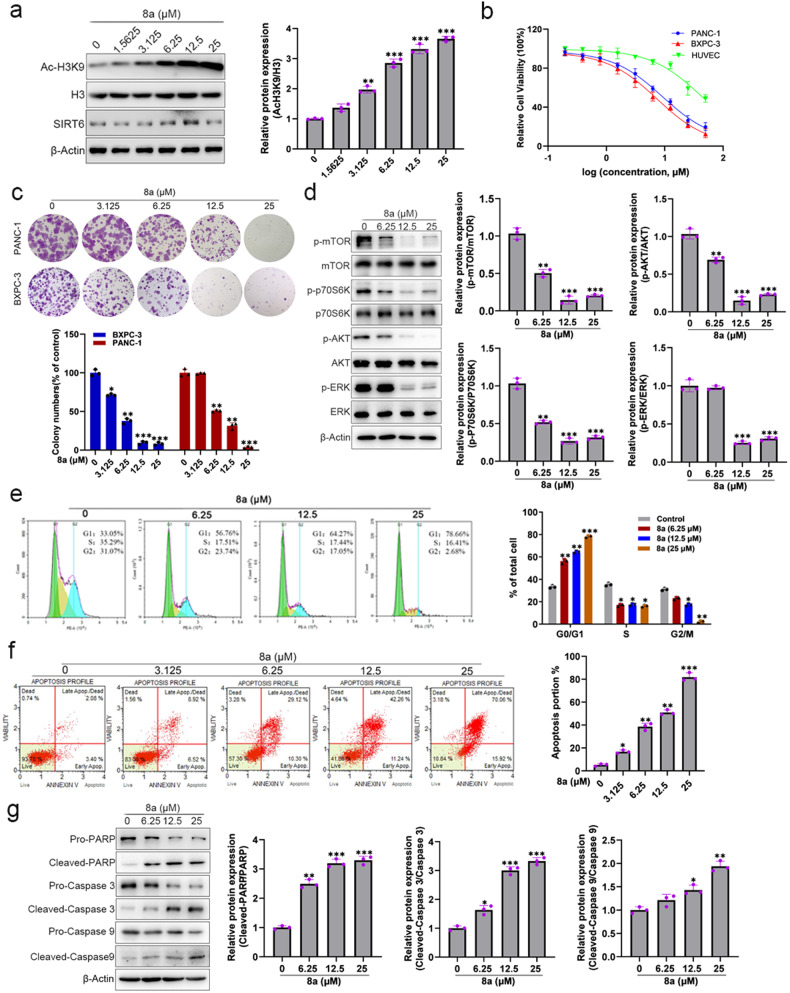
Compound 8a inhibits cell proliferation, induces cell-cycle arrest and apoptosis in PDAC cells. **a** BXPC-3 cells were treated with compound 8a. The expression of acetylated H3K9, H3, and SIRT6 were analyzed by western blot. **b** The cytotoxicity data of 8a in cancer cell lines and normal cell lines after treatment with 8a for 72 h. **c** 8a blocks colony formation of BXPC-3 cells, then the cells were stained with crystal violet and quantified. **d** The proliferation-related protein expression decreases after being given with 8a. **e** Flow cytometry detected the cell cycle of BXPC-3 cells and the percentage of cells. **f** Flow cytometry was applied to apoptosis of cancer cells and the percentage of cells. **g** The proteins expression of cleaved-PARP, cleaved-Caspase 3, and cleaved-Caspase 9 were increased following the 8a treatment. Data are presented as mean ± SD (*n* = 3). *P < 0.05, **P < 0.01, ***P < 0.001, versus control.

PDAC is a highly aggressive malignancy with limited effective therapeutic options [29]. We found that 8a increased the acetylation level of H3K9 and showed a dose-dependent effect in BXPC-3 cells (Fig. 3a), indication 8a could inhibit endogenous SIRT6 in PDAC cells. MTT assay indicated 8a significantly reduced the proliferation of BXPC-3 and PANC-1 cells with IC_50_ values at 72 h of 7.56 μM and 9.28 μM, respectively (Fig. 3b). But, 8a displayed low potency against normal HUVEC cells (IC_50_ = 41.01 μM) (Fig. 3b). Colony formation assays reflect the cell growth and survival abilities of a single cell. As shown in Fig. 3c, PDAC cells exhibited much smaller and fewer colonies after being treated with 8a in a dose-dependent manner.

The PI3K/AKT/mTOR and MAPK/ERK pathways are important positive regulators for the cell proliferation and survival [30, 31]. Previous studies showed that knockdown of SIRT6 can inhibit MAPK/ERK and PI3K/AKT/mTOR signaling pathways in hepatocellular carcinoma and diffuse large B-cell lymphoma [32, 33]. In this study, we investigated the effect of SIRT6 on PI3K/AKT/mTOR and MAPK/ERK pathways in BXPC-3 cells. As depicted in Fig. S3, the acetylation level of histone H3 was significantly increased after knockdown of SIRT6. The phosphorylation levels of AKT, mTOR, P70S6K and ERK proteins, were obviously decreased in SIRT6 knockdown cells compared to the negative control cells. Then, the effects of 8a on AKT/mTOR/P70S6K and ERK pathways were examined. As expected, p-mTOR, p-P70S6K, p-AKT, and p-ERK were significantly down-regulated after treatment with 8a (Fig. 3d). In addition, we also found that 8a could inhibit the activity of both mTORC1 and mTORC2 (Fig. S4). These findings suggested that 8a, as a SIRT6 inhibitor, may inhibit the proliferation of pancreatic cancer cells by inhibiting the PI3K/AKT/mTOR and ERK signaling pathways.

Next, we further examined the effects of 8a on PDAC cell cycle and apoptosis. After incubated with 8a for 48 h, 8a treatment increased the percentages of the G0-G1 phase (Fig. 3e) and decreased cyclin D1 expression (Fig. S5b) in a dose-dependent manner. Hoechst staining and flow cytometry were applied to investigate cells apoptosis. Hoechst staining showed a markedly shrunk of the chromatin with an increasing concentration of 8a (Fig. S5a). Flow cytometry analysis also showed that 8a induced BXPC-3 cells apoptosis in a dose-dependent manner (Fig. 3f). Subsequently, some apoptosis-associated factors, namely cleaved-PARP, cleaved-Caspase3, and cleaved-Caspase9, were determined by western blot. The results indicated that 8a significantly up-regulated the expression of cleaved-PARP, cleaved-Caspase3, and cleaved-Caspase9 (Fig. 3g). Altogether, these results demonstrated that 8a could inhibit PDAC cells proliferation, induces cell-cycle arrest and cell apoptosis.

### Compound 8a sensitizes PDAC cells to gemcitabine by inhibiting cell survival signaling

**Fig. 4:**
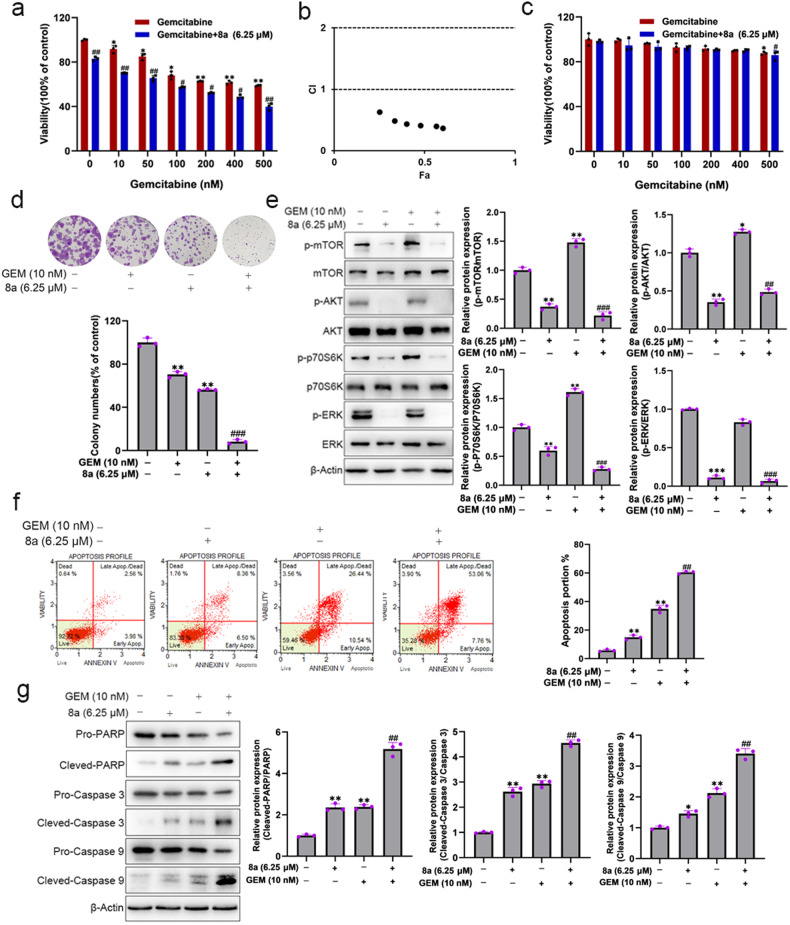
Compound 8a sensitizes pancreatic cancer cells to gemcitabine. **a** Cell viability of BXPC-3 cells after exposure to increasing concentration of gemcitabine in presence or absence of 6.25 μM 8a for 48 h. **b** CompuSyn software calculated viability data and plotted Fa-CI curves to analyze the synergistic effects of the two drugs. **c** Cell viability of HUVEC cells after exposure to increasing concentration of gemcitabine in presence or absence of 6.25 μM 8a for 48 h. **d** The colony formation assay of BXPC-3 cells after treatment with 10 nM gemcitabine, 6.25 μM 8a, or their combination for 10 days. **e** Western blotting analysis of proliferation-related protein expression. **f** Analysis of apoptosis by the Muse cell analyzer in BXPC-3 cells following exposure to gemcitabine or/and 8a. **g** Western blot analysis of cleaved-PARP, cleaved-Caspase 3, and cleaved-Caspase 9 in BXPC-3 cells after indicated treatment. Data are presented as mean ± SD (*n* = 3). *P < 0.05, **P < 0.01, ***P < 0.001, versus control. ^###^*P* < 0.001, versus GEM group.

Gemcitabine is a classic drug used for pancreatic cancer therapy, but chemoresistance reduces its therapeutic effect [2]. SIRT6-deficient cells demonstrate enhanced sensitivity to chemotherapeutic agents [14]. Thus, we evaluated the possibility that 8a can sensitize PDAC cells to gemcitabine. MTT and colony formation assay showed that the 8a significantly enhanced the effects of gemcitabine on the cell viability and clonogenic ability (Figs. 4a, d). We found synergistic effects of 8a and gemcitabine by combined effect analysis, with CI index less than 1 (Fig. 4b). In addition, we examined the cytotoxicity of 8a and gemcitabine to normal cells, and found that the combination of 8a and gemcitabine had weak cytotoxicity on HUVEC cells, much lower than that on PDAC cells (Fig. 4c).

To confirm the synergistic anti-PDAC activity, we performed the cell cycle and apoptosis assays. 8a significantly enhanced the G0/G1 arrest and S phase inhibition induced by gemcitabine (Fig. S6a). Consistently, the expression of cyclin D1 was also decreased following combination treatment (Fig. S6b). Hoechst staining (Fig. S6c) and flow cytometry assays (Fig. 4f) were used for apoptosis detection, the results showed that the combination of 8a and gemcitabine significantly increased the apoptosis of BXPC-3 cells compared to gemcitabine alone. Western blot analysis of apoptosis-related proteins, including cleaved-PARP, cleaved-Caspase 3, and cleaved-Caspase 9, showed that combination treatment induced much higher levels of these proteins than gemcitabine alone in BXPC-3 cells (Fig. 4g). Altogether, these results demonstrated the synergistic effects of 8a and gemcitabine on anti-PDAC cells.

Previous studies also showed that gemcitabine activates AKT and ERK signaling pathways in PDAC cells [34, 35], which may therefore impede the therapeutic efficacy of gemcitabine [34, 36]. In this study, we also found that gemcitabine alone increased phosphorylation levels of AKT and ERK, and 8a could reverse the activation of AKT and ERK signaling pathways induced by gemcitabine (Fig. 4e). Altogether, these results demonstrated the synergistic effects of 8a and gemcitabine on anti-PDAC cells, which may have been due to the 8a-mediated suppression of the AKT and ERK cell survival pathway, providing a possible mechanism for reversing chemoresistance of gemcitabine.

### Compound 8a enhances gemcitabine-induced DNA damage

**Fig. 5:**
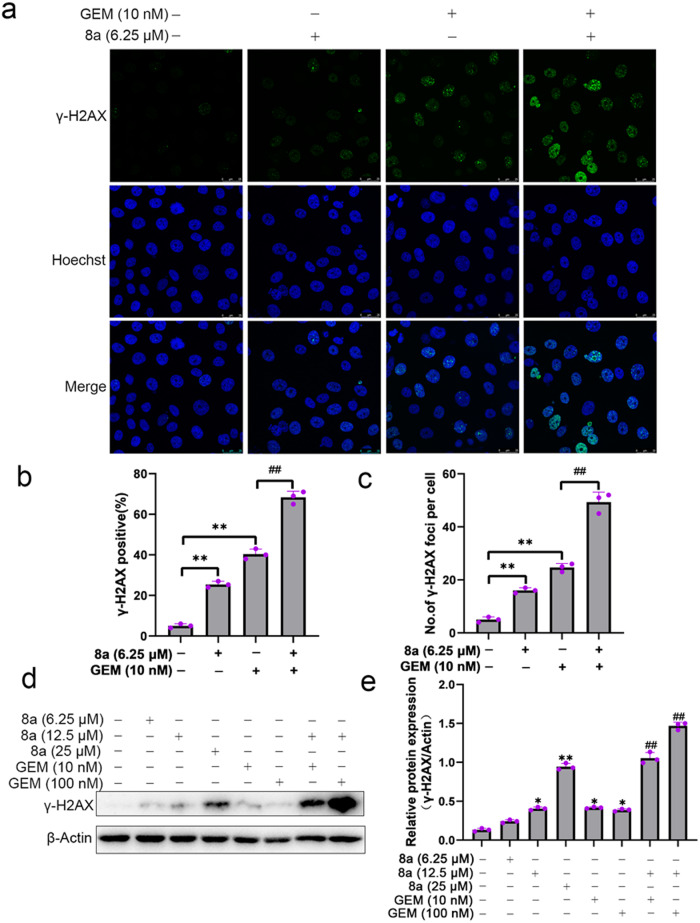
Compound 8a enhances gemcitabine-induced DNA damage. **a**–**c** γ-H2AX staining in BXPC-3 cells treated with 10 nM of gemcitabine, 6.25 μM of 8a or their combination for 24 h (scale bar: 25 μm). **d**, **e** Quantification of γ-H2AX protein expression after BXPC-3 cells treated with different doses of gemcitabine, 8a, or combination for 24 h by western blot. Data are presented as mean ± SD (*n* = 3). *P < 0.05, **P < 0.01, versus control. ^##^*P* < 0.01, versus GEM group.

Gemcitabine, a deoxycytidine nucleoside analog that inhibits DNA synthesis and induces cell death, shows its killing power mainly through the induction of DNA damage [2]. The presence of DNA repair pathways reduces tumor sensitivity to gemcitabine [37–39]. SIRT6 has been reported to be involved in regulating DNA repair [40]. Based on these studies, we hypothesized that SIRT6 inhibitor can lead to cancer cells sensitivity to DNA damaging agents in cancer chemotherapy by blocking DNA repair. γ-H2AX, phosphorylation of the Ser-139 residue of the histone variant γ-H2AX, which is a highly specific and sensitive molecular marker for monitoring DNA damage [41]. We found that gemcitabine can cause γ-H2AX foci, and its combination with 8a treatment can induce much more γ-H2AX foci (Fig. 5a–c). Consistent with the above results, western blot analysis also showed that the γ-H2AX expression levels were significantly enhanced when treatment with gemcitabine and 8a together (Fig. 5d, e). Collectively, these results suggested that inhibition of SIRT6 by 8a disturbed DNA repair activity and allowed accumulation of DNA damage resulted from gemcitabine and subsequently induced cell apoptosis.

### Compound 8a exhibited metabolic safety in vivo and in vitro

**Fig. 6:**
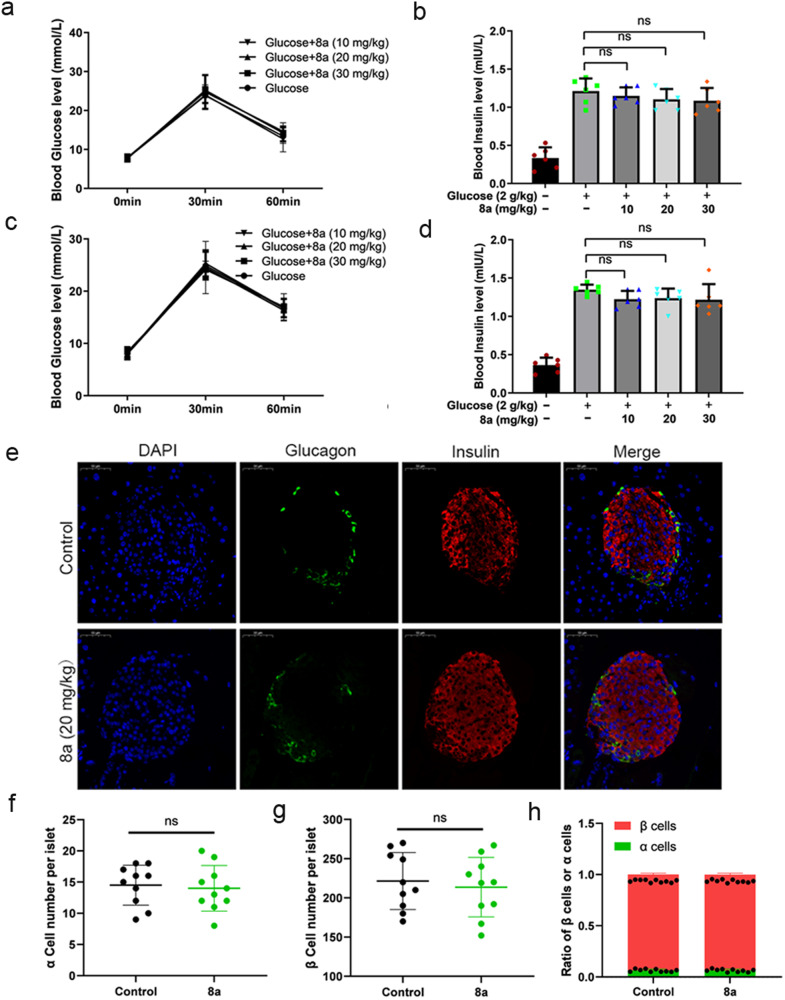
Compound 8a had no significant effect on plasma insulin levels and the composition of islet cells in mice. **a** Blood glucose levels in mice treated with 8a treated with 2 g/kg glucose for a short time. **b** Plasma insulin levels in mice treated with 8a for a short time. **c** Blood glucose levels in mice treated with 8a for a long time (2 weeks). **d** Plasma insulin levels in mice treated with 8a for a long time (2 weeks). **e** Representative immunofluorescent image of mouse pancreatic tissue after 2 weeks of 8a (20 mg/kg) treatment. Nuclei (DAPI), α-cells (Glucagon) and β-cells (insulin). Scale bar, 50 μm. Quantification of the islet α-cell number (**f**), β-cell number (**g**) and the ratio of β cells and α cells (**h**). Data are presented as mean ± SD (*n* = 6).

SIRT6 plays versatile roles in regulating metabolism, such as glucose homeostasis, insulin resistance, and many other processes [6, 7, 42–44]. Therefore, the SIRT6 inhibitor 8a may affect normal metabolic processes. We examined the effects of 8a on insulin signaling pathway and glucose uptake in islet β cells (β-TC-6) and pancreatic β-cells (NIT-1). We found the 8a treatment did not affect the elevation of insulin receptor phosphorylation levels induced by insulin (Fig. S7a–d), and the combination of 8a and insulin had no significant effect on glucose uptake compared to the insulin-only treatment to cells (Fig. S7e, f). In addition, we assessed the effect of 8a on pancreatic function in vivo. We examined the changes in glucose uptake capacity and glucose-stimulated insulin secretion levels in mice treated with 8a for short term and long term. The results showed that blood glucose and insulin concentration were no significant difference in the combined (8a + glucose) treatment group compared to the glucose group (Fig. 6a–d). We next evaluated the effects of 8a on islet cell composition. We found that there was no significant difference in the number of α cells and β cells in the islets after 8a treatment (Fig. 6e–h). Collectively, these results indicate that 8a had no significant metabolic toxicity in vivo and in vitro.

### Compound 8a augmented the antitumor effects of gemcitabine in BXPC-3 tumor xenografts

**Fig. 7:**
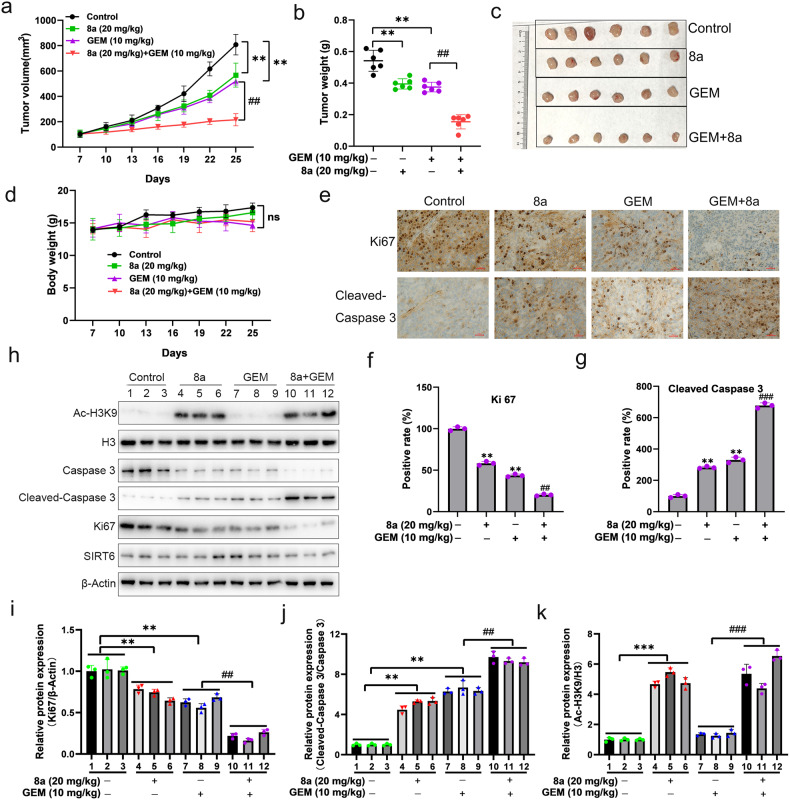
Combination of 8a and gemcitabine inhibits pancreatic cancer growth in vivo. **a**–**c** Mice bearing BXPC-3 xenografts were treated with vehicle control, 8a (20 mg/kg), gemcitabine (10 mg/kg), or their combination. Tumor growth curve (**a**), tumor weight (**b**), and visible tumor formation (**c**) were shown. **d** Body weight changes of mice during treatment. **e**–**g** Ki67 and cleaved-Caspase 3 were analyzed in tumor tissues at the end of experiments by immunohistochemical staining (scale bar: 100 μm). **h**–**k** Representative western blots of Ac-H3K9, H3, pro-Caspase 3, cleaved-Caspase 3, Ki67, SIRT6 and β-Actin levels in three representative BXPC-3 xenograft tumors from each group in vivo. Data presented as mean ± SD of six mice per group. **P < 0.01, ***P < 0.001, versus control. ^##^*P* < 0.01, ^###^*P* < 0.001, versus GEM group.

To further confirm whether 8a can enhance the antitumor effects of gemcitabine in vivo, the BXPC-3 PDAC xenografts were used. The mice with tumors received intraperitoneal injection of compound 8a (20 mg/kg), gemcitabine (10 mg/kg), or their combination once every 2 days for 4 weeks. Tumor volumes and body weight of mice were monitored every 3 days. As shown in Fig. 7a–c, the compound 8a only had 27% tumor inhibition compared with the control mice, and the mice treated with gemcitabine could inhibit tumor mass by 30.1%. However, the tumor mass was inhibited by up to 71.3% in mice treated with the combinations of 8a and gemcitabine. Notably, mice in different treatment groups showed no significant loss of body weight compared with the control group (Fig. 7d). In addition, immunohistochemistry analysis showed that the combinations of 8a and gemcitabine greatly increased the expression of apoptosis maker, activated Caspase 3, and reduced the expression of proliferation marker, Ki67 (Fig. 7e–g). Western blotting of the tumor tissue samples showed that 8a treatment elevated the Ac-H3K9, and combinations of 8a and gemcitabine increased cleaved-Caspase three levels, but decreased the Ki67 expression levels, which were consistent with the immunohistochemistry results (Fig. 7h–k). These results showed that 8a significantly enhanced gemcitabine antitumor effects in vivo by inhibition tumor cell proliferation and induction of apoptosis.

## Discussion

SIRT6 has been confirmed to be involved in a variety of biological processes, such as gene transcription, genome stability and DNA repair [8]. SIRT6 also plays important roles in multiple diseases, including metabolic diseases, aging and cancer [8, 45]. Some studies suggested that overexpression of SIRT6 induced resistance to antitumor therapy by mediating the DNA repair [33, 46]. Although SIRT6 has emerging potential as a therapeutic target in cancer, few small molecule inhibitors of SIRT6 have been identified to date, and most of these inhibitors show weak inhibitory activity against SIRT6 and limited antitumor activity [45, 47]. In this study, we identified a new non-competitive SIRT6 inhibitor compound 8a with an IC_50_ of 7.46 ± 0.79 μM. Currently, OSS_128167, the only commercially available SIRT6 inhibitor, shows a SIRT6 inhibitory activity with an IC_50_ of 89 μM [16]. Additionally, SIRT6 inhibitors (compounds 1, 3 and 8) with a quinazolinedione structure were found with an IC_50_ of 106 ± 16, 37 ± 2 and 49 ± 4 μM, respectively [17]. Compared with these inhibitors, 8a showed the most potent SIRT6 inhibitory activity.

Previous studies have shown that SIRT6 inhibitors have the ability to regulate cell proliferation, migration and invasion of DLBCL and TNBC [33, 48]. In this study, we demonstrated that 8a can inhibit cell proliferation, and induce cell cycle arrest and apoptosis in PDAC cells. The PI3K/AKT/mTOR and MAPK/ERK pathways are important regulators for the cell proliferation and survival [30, 31]. Our data demonstrated that 8a could inhibit PI3K/AKT/mTOR and ERK signaling pathway, which was consistent with SIRT6 knockout effect in PDAC cells, suggesting 8a might inhibit PDAC cells proliferation and survival by inhibiting PI3K/AKT/mTOR and ERK signal pathways.

Gemcitabine is the first-line chemotherapy for pancreatic cancer [2]. However, high rate of chemoresistance severely impedes its efficacy as a cornerstone of pancreatic cancer chemotherapy [2, 3, 49]. We found that the combination of 8a and gemcitabine synergistically played antitumor roles in PDAC cells. It has been reported that gemcitabine activates AKT and ERK signaling pathways to cause resistance in pancreatic cancer [34, 35]. Several agents including PI3K inhibitor (LY294002) or AKT inhibitor (MK2206) have been demonstrated alone or in combinations with DNA-targeted drugs (gemcitabine and 5-fluorouracil) in pancreatic cancer [34, 50]. Our data suggested that 8a reversed the activation of AKT and ERK signaling pathways induced by gemcitabine and augment the efficacy of gemcitabine. Therefore, the ability of 8a to inhibit AKT and ERK signaling pathways may be an important reason for its synergistic effect with gemcitabine.

The nucleoside analog gemcitabine inhibits DNA synthesis and ribonucleotide reductase, therefore inducing significant DNA damage and causing cancer cell apoptosis [39]. However, the existence of DNA repair pathways reduces cancer cell sensitivity to gemcitabine [37–39]. SIRT6 has been described to be involved in regulating DNA repair [40]. Therefore, we evaluated the possibility that SIRT6 inhibitors may be involved in chemoresistance. Our results suggested that 8a impaired DNA repair activity and allowed accumulation of DNA damage resulted from gemcitabine and subsequently induced cell apoptosis. These results were consistent with other reports that SIRT6 inhibitors sensitize cancer cells to gemcitabine via inducing DNA damage and apoptosis [15, 17]. More importantly, this study demonstrated that small molecule SIRT6 inhibitors promote antitumor drug sensitivity in vivo tumor models for the first time. Our result also suggests that SIRT6 inhibitor might be helpful for improving sensitivity of DNA-target chemotherapeutic agents in cancer and we might conduct more in-depth research on this aspect in the future.

In conclusion, we discovered and synthesized a potent SIRT6 inhibitor compound 8a, which exhibited considerable anti-PDAC activities and enhances sensitivity of PDAC to gemcitabine in vitro and in vivo by inhibiting cell proliferation and survival signaling pathways, including AKT/mTOR/P70S6K and ERK signaling pathways, and enhancing DNA damage induced by gemcitabine. Overall, 8a could be a promising therapeutic candidate for sensitizing pancreatic cancer to gemcitabine.

## Supplementary information

Supplementary information

Western Blot

A reproducibility checklist

## Data Availability

All data generated and analyzed during this study are included in this article and its Supplementary Information files. Additional data are available from the corresponding author on reasonable request.
