# Supplementary material for: Discovery of a pyrrole-pyridinimidazole derivative as novel SIRT6 inhibitor for sensitizing pancreatic cancer to gemcitabine
Source: Cell Death Dis. 2023 Aug 4;14(8):499. doi: 10.1038/s41419-023-06018-1 (PMC10403574; doi:10.1038/s41419-023-06018-1)

**Supporting information**

**Discovery of a Pyrrole-pyridinimidazole Derivative as Novel SIRT6 Inhibitor for** **Sensitizing Pancreatic Cancer to Gemcitabine**

Nannan Song ^a,#^, Xian Guan ^a,#^, Siqi Zhang ^a^, Yanqing Wang ^a^, Xuekai Wang ^a^, Zhongxia Lu ^a^, Daochen Chong ^c^, Jennifer Yiyang Wang ^a^, Rilei Yu ^a, b^,Wengong Yu ^a, b,*^, Tao Jiang ^a,^ ^b, *^, and Yuchao Gu ^a, b, *^

^a^ Key Laboratory of Marine Drugs, Ministry of Education, School of medicine and Pharmacy, Ocean University of China, Qingdao 266003, P.R. China

^b^ Pilot National Laboratory for Marine Science and Technology (Qingdao), Qingdao 266237, China

^c^ Department of Pathology, 971 Hospital of PLA Navy, Qingdao 266071, China.

**This file includes:**

**Table S1.** Target selectivity of 8a among HDACs and sirtuins.

**Figure S1.** Compound 8a is a non-competitive inhibitor of SIRT6.

**Figure S2.** Effect of 8a on deacetylation activity of SIRT6 mutants, as determined by FDL assays.

**Figure S3.** Knockdown of SIRT6 inhibited activation of PI3K/AKT/mTOR and MAPK/ERK pathways in PDAC cells.

**Figure S4.** Compound 8a could inhibit the activities of mTORC1 and mTORC2.

**Figure S5.** Compound 8a induces cell-cycle arrest and apoptosis in PDAC cells.

**Figure S6.** Compound 8a greatly potentiates gemcitabine-induced cell cycle arrest and apoptosis.

**Figure S7.** Compound 8a had no significant effect on glucose uptake capacity in different groups of 8a in NIT-1 and β-TC-6 cells.

**Chemistry** General procedure to synthesis compounds, Copies of ^1^H and ^13^C NMR spectra of the final compounds and Copies of HRMS (ESI) spectra of compounds 5c, 6b and 8a.

**Table S1. Target selectivity of 8a among HDACs and sirtuins.**

| Enzyme | IC_50_ (*μ*M) * | Enzyme | IC_50_ (*μ*M) * |
| --- | --- | --- | --- |
| SIRT1 | 80.52 ± 1.91 | HDAC4 | ＞200 |
| SIRT2 | 92.21 ± 1.95 | HDAC5 | ＞200 |
| SIRT3 | ＞200 | HDAC6 | 96.77 ± 1.98 |
| SIRT5 | ＞200 | HDAC7 | ＞200 |
| SIRT6 | 7.46 ± 0.79 | HDAC8 | 102 ± 2.01 |
| HDAC1 | ＞200 | HDAC9 | ＞200 |
| HDAC2 | ＞200 | HDAC10 | ＞200 |
| HDAC3 | 111.9 ± 2.05 | HDAC11 | ＞200 |

*The activity values are calculated based on IC_50_ for the inhibition effect. Data are presented as the mean ± SD from three independent experiments.


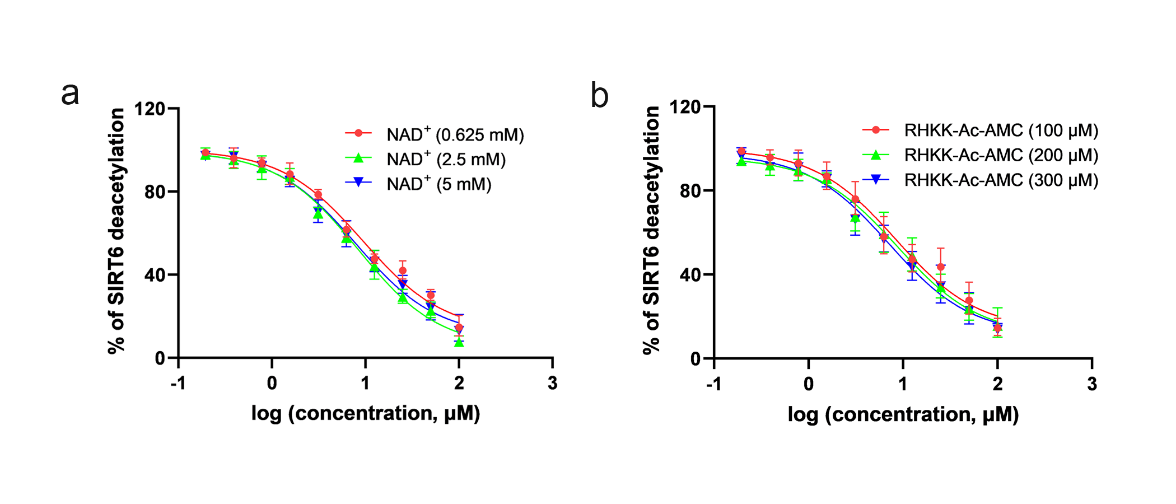


**Figure S1.** **Compound 8a is a non-competitive inhibitor of SIRT6.** (**a**) Competition assay between NAD^+^ and 8a, as evaluated by the Fluor-De-Lys (FDL) assay. (**b**) Competition assay between RHKK-Ac-AMC and 8a, as evaluated by the Fluor-De-Lys (FDL) assay. Data are presented as the mean ± SD, n = 3 wells, from three independent experiments.

**Figure S2.** **Effect of 8a on deacetylation activity of SIRT6 mutants, as determined by FDL assays.** Data are presented as the mean ± SD, n = 3 wells, from three independent experiments.


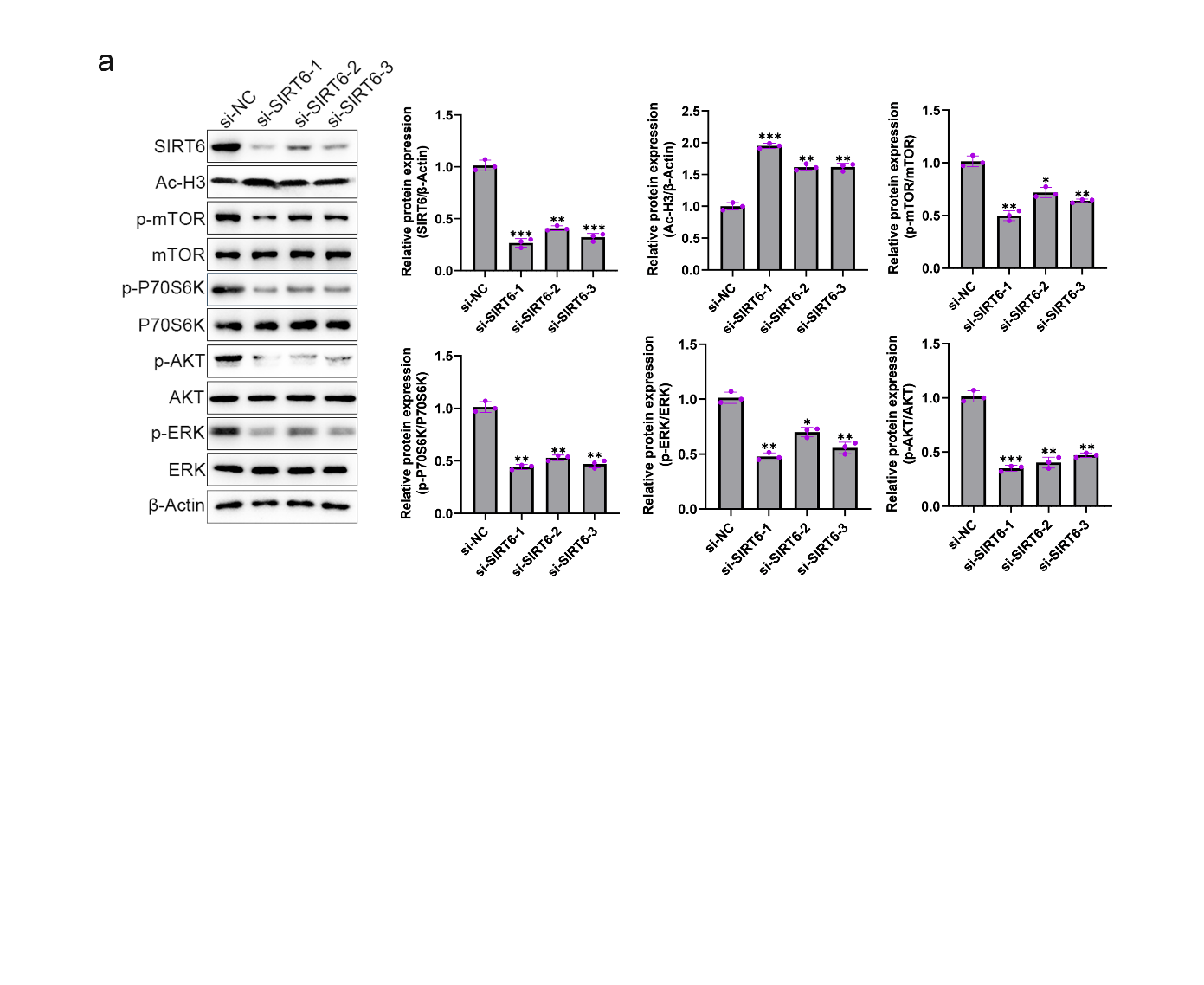


**Figure S3. Knockdown of SIRT6 inhibited activation of PI3K/AKT/mTOR and MAPK/ERK pathways in PDAC cells.** Western blot analysis of SIRT6, Ac-H3, p-AKT, p-mTOR, p-P70S6K and p-ERK in BXPC-3 cells at 72 h post-transfection with NC or si-SIRT6. Data are presented as mean ± SD (n=3). *P < 0.05, **P < 0.01, ***P < 0.001, versus si-NC.


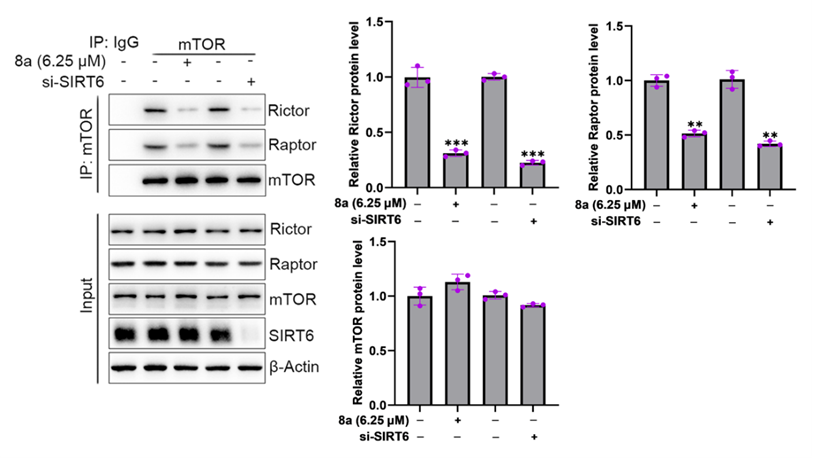


**Figure S4. Compound 8a could inhibit the activities of mTORC1 and mTORC2.** BXPC-3 cells were treated with 8a or knocked down for SIRT6, and mTOR was immunoprecipitated. Rictor, Raptor and mTOR were analyzed by immunoblotting with the indicated antibodies. The cell lysates were subjected to direct immunoblot with same antibodies (Input).


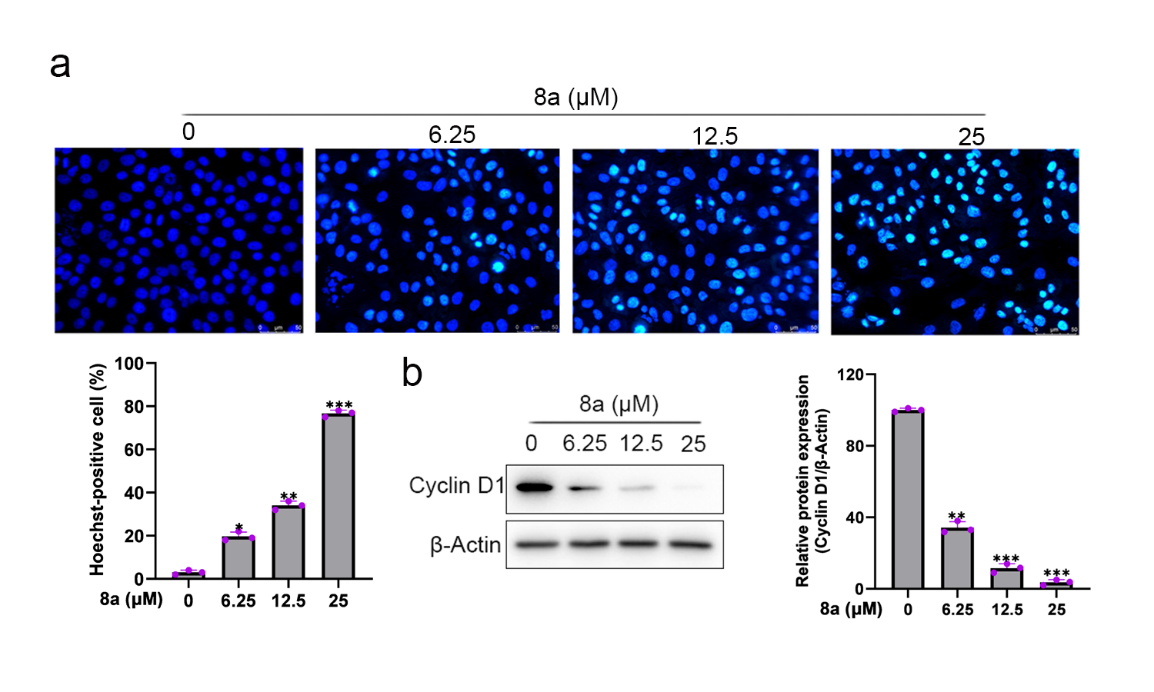


Figure S5. Compound 8a induces cell-cycle arrest and apoptosis in PDAC cells. (a) Hoechst staining was applied to apoptosis of cancer cells and the percentage of cells (scale bar: 50 *μ*m). (b) The protein expression of cyclin D1 was decreased following the 8a treatment. Data are presented as mean ± SD (n=3). *P < 0.05, **P < 0.01, ***P < 0.001, versus control.


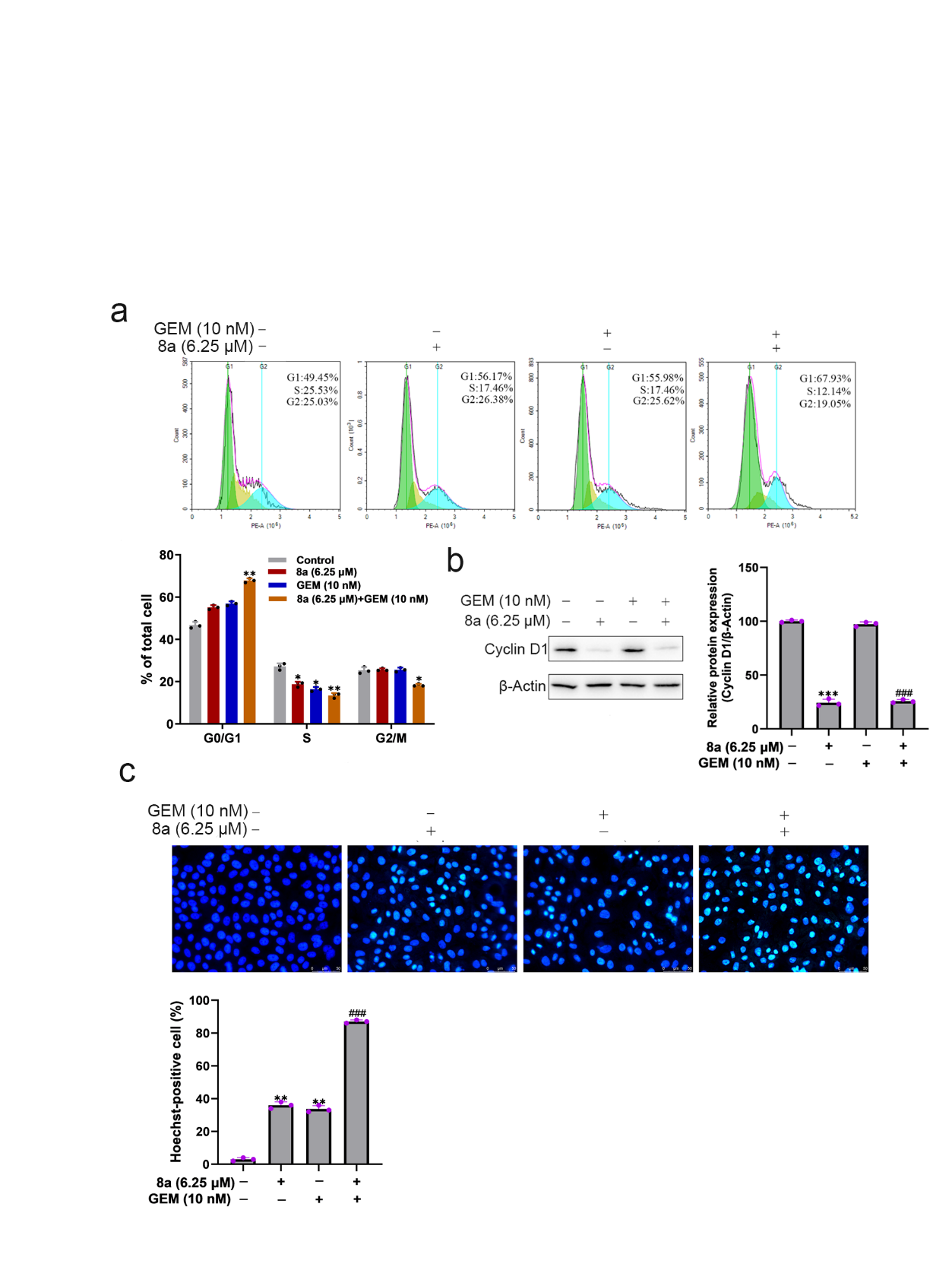


**Figure S6.** **Compound 8a greatly potentiates gemcitabine-induced cell cycle arrest and apoptosis.** (**a**) Flow cytometry analysis of cell cycle propidium iodide (PI) staining in BXPC-3 cells after treated with the combination or alone. (**b**) Analysis of cyclin D1 expression in cancer cells with indicated treatment for 48 h using western blot assay. (**c**) Compound **8a** increased the number of apoptotic vesicles in BXPC-3 cells induced by gemcitabine with Hoechst staining (scale bar: 50 *μ*m). Data are presented as mean ± SD (n=3). **P < 0.01, ***P < 0.001, versus control. ^###^P < 0.001, versus GEM group.


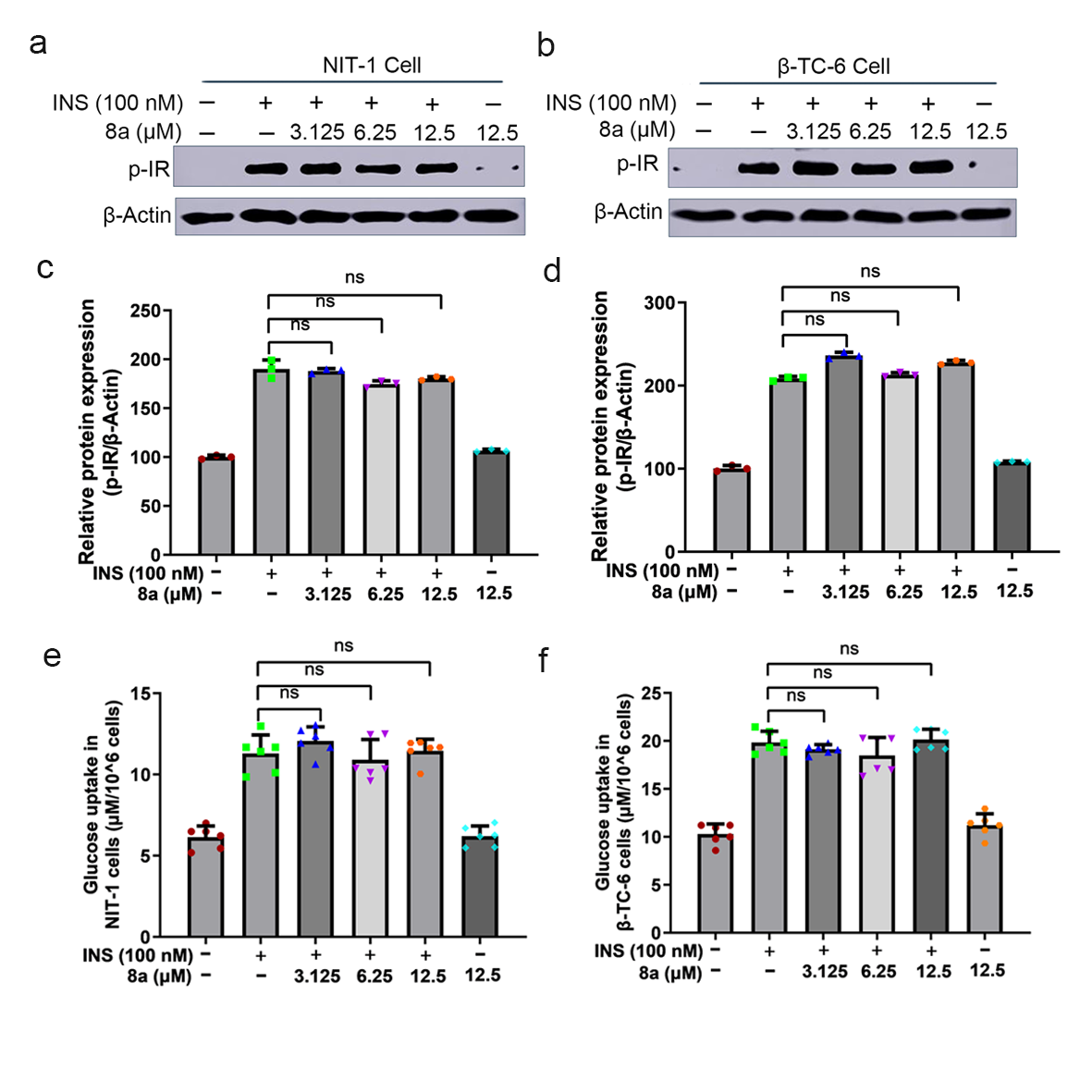


**Figure S7.** **Compound 8a had no significant effect on glucose uptake capacity in different groups of 8a in NIT-1 and β-TC-6 cells**. (a, c) The effect of 8a on insulin receptor phosphorylation in NIT-1 cells. (b, d) The effect of 8a on insulin receptor phosphorylation in β-TC-6 cells. (e) The effect of 8a on glucose uptake capacity in NIT-1 cells. (f) The effect of 8a on glucose uptake capacity in β-TC-6 cells. Data are presented as mean ± SD (n=3).

**Chemistry**

All commercially available starting materials and solvents were purchased from commercial vendors and used without further purification. Reactions were monitored using analytical thin-layer chromatography (TLC) on precoated silica gel GF254 plates (Qingdao Haiyang Chemical Plant, Qingdao, China) and visualized under ultraviolet light (254 nm and 365 nm). Column chromatography was performed on silica gel (200 - 300 mesh). ^1^H and ^13^C NMR spectra were recorded on the Broker AVANCE NEO and Agilent DD2 500 with 400 or 500 MHz for proton (^1^H NMR) and 100 or 125 MHz for carbon (^13^C NMR) with tetramethylsilane (Me_4_Si) as the internal standard, respectively. The chemical shifts (*δ*) were expressed in parts per million (ppm). Abbreviations used: s = singlet; d = doublet; t = triplet; q = quartet; m = multiplet. The coupling constant (*J*) values were described as hertz. High-resolution (ESI) MS spectra were recorded using a QTOF-2 Micromass spectrometer. The purity of the final compounds for biological evaluation was higher than 95% by analytical HPLC analysis with the Primaide 1210 system.

Compounds **2**, **3 - 5**, and **6 - 8** were prepared according to the procedure published by Zhong *et al,* and Naoki *et al,* the spectroscopic data for the intermediates were identical to those described in the literature^66^.

*General Procedure for Compounds* ***6-8****.* To the solution of **3** - **5** (0.1 mmol) and histamine dihydrochloride (1.5 mmol) and triethylamine (3.5 mmol), in EtOH was stirred at 80 °C for 12 h, respectively. The mixture was concentrated in vacuo, and the residue was purified by column chromatography (dichloromethane/methanol 10:1) to give the compounds **6** - **8** as a yellow amorphous solid with a yield of 40% or so.

*General Procedure for Compounds* ***1a - 1c****.* To a solution of **6** - **8** (1 mmol) in DMSO was added IBX (1.5 mmol), and the mixture was stirred at room temperature for 2 h. The reaction was quenched by adding H_2_O and an aqueous NaOH solution (1.0 mol/L, 300 mL), and the mixture was extracted with EtOAc. The organic extracts were combined, washed with H_2_O and brine successively, dried over anhydrous MgSO_4_, filtered, and then concentrated in vacuo. To a solution of the residue in CH_2_Cl_2_ was added activated MnO_2_ (5.0 mol), and the mixture was stirred at room temperature for 2.5 h. The reaction mixture was filtered, and the filtrate was concentrated in vacuo. The residue was purified by column chromatography (petroleum ether/ethyl acetate 4:1) to give **1a** - 1**c**.

*4-[4,5-dibromo-1-[[2-(trimethylsilyl)ethoxy]methyl]-1H-pyrrol-2-yl]-3H-imidazo[4,5-c]pyridine (****1a****)*. Yellow powder (235mg, 50%); ^1^H NMR (500 MHz, DMSO-*d*_6_) *δ* 8.45 (s, 1H), 8.32 (d, *J* = 5.5 Hz, 1H), 7.57– 7.46 (m, 2H), 6.13 (s, 2H), 3.24 (t, *J* = 7.9 Hz, 2H), 0.59 (t, *J* = 8.0 Hz, 2H), -0.30 (s, 9H);^13^C NMR (125 MHz, DMSO-*d*_6_) *δ* 144.50, 140.39, 131.82, 117.24, 109.99, 109.35, 100.03, 75.29, 65.17, 17.45, -1.25. HRMS (ESI+) *m/z*: Calcd for C_16_H_20_Br_2_N_4_OSi; [M+H]^+^ 469.9773, Found 470.9846.

*4-[1-[[2-(trimethylsilyl)ethoxy]methyl]-1H-pyrrol-2-yl]-3H-imidazo[4,5-c]pyridi*

*ne (****1b****).* Yellow powder (158 mg, 50%);^1^H NMR (400 MHz, Chloroform-*d*) *δ* 8.34 (d, *J* = 5.6 Hz, 1H), 8.08 (s, 1H), 7.43 (d, *J* = 4.6 Hz, 1H), 7.08 (s, 1H), 7.03 (dd, *J* = 2.7, 1.7 Hz, 1H), 6.36 (d, *J* = 2.9 Hz, 1H), 5.83 (s, 2H), 3.47 – 3.45 (m, 2H), 0.84 – 0.78 (m, 2H), -0.14 (s, 9H);^13^C NMR (100 MHz, Chloroform-*d*) *δ* 143.02, 139.97, 128.41, 126.27, 109.65, 66.08, 29.72, 17.79, 15.30, -1.58. HRMS (ESI+) *m/z*: Calcd for C_16_H_22_N_4_OSi; [M+H]^+^ 314.1563, Found 315.1639.

*4-(4,5-dibromo-1-methyl-1H-pyrrol-2-yl)-1H-imidazo[4,5-c]pyridine (****1c****)**.* Yellow powder (140 mg, yield 40%); ^1^H NMR (400 MHz, Methanol-*d*_4_) *δ* 8.40 (d, *J* = 5.7 Hz, 1H), 8.38 (s, 1H), 7.62 (d, *J*= 5.7 Hz, 1H), 6.94 (s, 1H), 3.87 (s, 3H); ^13^C NMR (101 MHz, Methanol-*d*_4_) *δ* 144.38, 144.30, 140.25, 130.42, 129.46, 114.84, 114.18, 108.37, 98.10, 34.88; HRMS (ESI^+^) *m/z*: Calcd for C_11_H_8_Br_2_N_4_; [M+H]^+^ 354.9205, Found 353.9116.

*4-(4,5-dibromo-1H-pyrrol-2-yl)-1H-imidazo[4,5-c]pyridine -TFA* *(****2a****).* To a solution of **1a** (0.20 mmol) in CH_2_Cl_2_ (5.0 mL) was added BF_3_-OEt_2_ (2.1 mmol) under a nitrogen atmosphere, and the mixture was stirred at room temperature for 15 h. The reaction was quenched by adding saturated sodium bicarbonate solution, and the reaction mixture was extracted with EtOAc. The combined extracts were washed with H_2_O and brine, dried over anhydrous MgSO_4_, filtered, and then concentrated in vacuo. The residue was dissolved in MeOH and TFA (0.10 mL) was added to the MeOH solution. The acidic methanolic solution was concentrated in vacuo. Trituration of the residue with CH_2_Cl_2_ gave **2a**. Yellow powder (77 mg, yield 85%); ^1^H NMR (500 MHz, DMSO-*d_6_*) *δ* 13.29 (s, 1H),8.73 (s, 1H), 8.32 (d, *J* = 6.2 Hz, 1H), 7.82 (s, 1H), 7.73 (d, *J* = 6.2 Hz, 1H); ^13^C NMR (125 MHz, DMSO-*d*_6_) *δ* 158.79, 158.53, 147.50, 142.36, 134.66, 118.91, 107.82, 100.88; HRMS (ESI^+^) *m/z*: Calcd for C_10_H_6_Br_2_N_4_; [M+H]^+^ 341.9940, Found 342.9020.

*4-(1H-pyrrol-2-yl)-1H-imidazo[4,5-c] pyridine -TFA* *(****2b****).* Yellow powder (53 mg, yield 87%); HRMS (ESI^+^) *m/z*: Calcd for C_10_H_8_N_4_; [M+H]^+^ 184.2020, Found 185.0827

*General Procedure for Compounds* ***3a - 3g****.* To a stirred solution of **1a** - 1**c** (0.75 mmol) in DMF (30 mL) was added Cs_2_CO_3_ (2.25 mmol) and appropriate dibromoalkyl (2.25mmol) at room temperature for 3 h. The reaction mixture was extracted with EtOAc. The combined extracts were washed with H_2_O and brine, dried over anhydrous MgSO_4_, filtered, and then concentrated in vacuo. The reaction residue was subjected to preliminary purification on a silica gel column (petroleum ether/ethyl acetat 8:1) to obtain **3a** - **3g**.

*1-(4-bromobutyl)-4-(4,5-dibromo-1-((2-(trimethylsilyl)ethoxy)methyl)-1H-pyrrol-2-yl)-1H-imidazo[4,5-c]pyridine* *(****3a****).* Yellow adhesive liquid (250 mg, yield 55%); ^1^H NMR (500 MHz, Chloroform-*d*) *δ* 8.42 (d, *J* = 5.6 Hz, 1H), 7.95 (s, 1H), 7.52 (s, 1H), 7.25 (d, J = 5.6 Hz, 1H), 6.18 (s, 2H), 4.25 (t, 2H), 3.43 (t, *J* = 15.4 Hz, 2H), 3.37 (t, *J* = 16.2 Hz, 2H), 2.12– 2.07 (m, 2H), 1.93 – 1.88 (m, 2H), 0.74 (t, *J* = 16.2 Hz, 2H), -0.23 (s, 9H); ^13^C NMR (126 MHz, CDCl_3_ ) *δ* 143.52, 143.36, 140.98, 139.28, 137.70, 118.23, 109.64, 103.61, 100.81, 75.40, 65.44, 44.48, 32.51, 32.22, 30.93, 29.68, 29.48, 28.50,17.63, -1.63; HRMS (ESI^+^) *m/z*: Calcd for C_20_H_27_Br_3_N_4_OSi; [M+H]^+^ 607.2660, Found 608.9547.

*1-(5-bromopentyl)-4-(4,5-dibromo-1-((2-(trimethylsilyl)ethoxy)methyl)-1H-pyrrol-2-yl)-1H-imidazo[4,5-c]pyridine* *(****3b****).* Yellow adhesive liquid (231 mg, yield 50%); ^1^H NMR (400 MHz, DMSO-*d*_6_) *δ* 8.46 (s, 1H), 8.35 (d, *J* = 5.6 Hz, 1H), 7.65 (d, *J* = 5.6 Hz, 1H), 7.54 (s, 1H), 6.17 (s, 2H), 4.31 (t, *J* = 7.0 Hz, 2H), 3.49 (s, 2H), 3.29 – 3.21 (m, 2H), 1.83 (p, *J* = 7.0 Hz, 4H), 1.36 (p, *J* = 7.8, 7.3 Hz, 2H), 0.65 – 0.58 (m, 2H), -0.30 (s, 9H); ^13^C NMR (100 MHz, DMSO-*d*_6_) *δ* 146.70, 142.96, 141.29, 140.56, 138.35, 132.64, 118.37, 110.22, 106.44, 100.76, 76.08, 65.88, 46.37, 45.37, 36.08, 32.80, 29.71, 25.83, 18.20, -0.50; HRMS (ESI^+^) *m/z*: Calcd for C_21_H_29_Br_3_N_4_OSi; [M+H]^+^ 619.9640, Found 620.9741.

*1-(6-bromohexyl)-4-(4,5-dibromo-1-((2-(trimethylsilyl)ethoxy)methyl)-1H-pyrrol-2-yl)-1H-imidazo[4,5-c]pyridine* *(****3c****)**.* Yellow adhesive liquid (282 mg, yield 60%); ^1^H NMR (500 MHz, Chloroform-*d*) *δ* 8.42 (d, *J* = 5.6 Hz, 1H), 7.95 (s, 1H), 7.52 (s, 1H), 7.24 (d, *J* = 5.6 Hz, 1H), 6.19 (s, 2H), 4.20 (t, *J* = 7.1 Hz, 2H), 3.41 – 3.34 (m, 4H), 1.92 (dt, *J* = 15.0, 7.3 Hz, 2H), 1.86 (dd, *J* = 14.3, 7.3 Hz, 2H), 1.51 (dd, *J* = 15.4, 7.7 Hz, 2H), 1.38 (dd, *J* = 15.4, 8.0 Hz, 2H), 0.75 – 0.70 (m, 2H), -0.23 (s, 9H); ^13^C NMR (125 MHz, Chloroform-*d*) *δ* 143.53, 140.81, 139.37, 137.73, 131.01, 118.22, 109.61, 103.67, 100.82, 75.40, 65.43, 45.12, 33.41, 32.30, 29.74, 27.49, 25.90, 17.62, -1.65; HRMS (ESI^+^) *m/z*: Calcd for C_22_H_31_Br_3_N_4_OSi; [M+H]^+^ 635.3140, Found 636.9863.

*1-(7-bromoheptyl)-4-(4,5-dibromo-1-((2-(trimethylsilyl)ethoxy)methyl)-1H-pyrrol-2-yl)-1H-imidazo[4,5-c]pyridine* *(****3d****).* Yellow adhesive liquid (273 mg, yield 55%); ^1^H NMR (400 MHz, DMSO-*d*_6_) *δ* 8.46 (s, 1H), 8.35 (d, *J* = 5.6 Hz, 1H), 7.65 (d, *J* = 5.6 Hz, 1H), 7.52 (s, 1H), 6.17 (s, 2H), 4.29 (t, *J* = 7.0 Hz, 2H), 3.49 (t, *J* = 6.7 Hz, 2H), 3.26 – 3.20 (m, 2H), 1.77 (m, *J* = 13.9, 7.0 Hz, 4H), 1.27 (d, *J* = 32.4 Hz, 6H), 0.63 – 0.57 (m, 2H), -0.32 (s, 9H); ^13^C NMR (100 MHz, DMSO-*d*_6_) *δ* 141.28, 140.67, 138.40, 132.64, 118.40, 110.27, 106.49, 100.79, 76.08, 65.89, 45.55, 36.37, 33.31, 30.55, 28.76, 28.59, 27.07, 18.21; HRMS (ESI^+^) *m/z*: Calcd for C_23_H_33_Br_3_N_4_OSi; [M+H]^+^ 649.3410, Found 651.0019.

*1-(8-bromooctyl)-4-(4,5-dibromo-1-((2-(trimethylsilyl)ethoxy)methyl)-1H-pyrrol-2-yl)-1H-imidazo[4,5-c]pyridine* *(****3e****).* Yellow adhesive liquid (250 mg, yield 50%); ^1^H NMR (400 MHz, DMSO-*d*_6_) *δ* 8.45 (s, 1H), 8.35 (d, *J* = 5.6 Hz, 1H), 7.63 (d, *J* = 5.6 Hz, 1H), 7.53 (s, 1H), 6.17 (s, 2H), 4.29 (t, *J* = 7.0 Hz, 2H), 3.48 (t, *J* = 6.7 Hz, 2H), 3.28 – 3.22 (m, 2H), 1.83 – 1.70 (m, 4H), 1.36 – 1.18 (m, 10H), 0.65 – 0.57 (m, 2H), -0.31 (s, 9H); ^13^C NMR (100 MHz, DMSO-*d*_6_) *δ* 147.28, 140.98, 138.37, 118.60, 110.55, 106.75, 100.90, 76.26, 65.96, 46.57, 45.68, 36.35, 33.36, 33.18, 30.58, 29.54, 29.31, 29.17, 28.61, 27.36, 27.12, 18.19, -0.5; HRMS (ESI^+^) *m/z*: Calcd for C_24_H_35_Br_3_N_4_OSi; [M+H]^+^ 663.3680, Found 665.0179.

*1-(4-bromobutyl)-4-(4,5-dibromo-1-methyl-1H-pyrrol-2-yl)-1H-imidazo[4,5-c]pyridine (****3f****).* Yellow adhesive liquid (220 mg, yield 60%); ^1^H NMR (400 MHz, DMSO-*d*_6_) *δ* 8.46 (s, 1H), 8.35 (d, *J* = 5.6 Hz, 1H), 7.62 (d, *J* = 5.6 Hz, 1H), 7.54 (s, 1H), 4.34 (t, *J* = 7.0 Hz, 2H), 4.07 (s, 3H), 3.55 (t, *J* = 6.5 Hz, 2H), 1.91 (q, *J* = 7.3 Hz, 2H), 1.78 (q, *J* = 7.0 Hz, 2H); ^13^C NMR (100 MHz, DMSO-*d*_6_) *δ* 145.78, 142.37, 140.63, 139.72, 137.41, 131.35, 116.72, 109.42, 105.36, 98.03, 44.01, 36.95, 34.75, 29.83, 28.64; HRMS (ESI^+^) *m/z*: Calcd for C_15_H_15_Br_3_N_4_; [M+H]^+^ 489.8826, Found 490.8899.

*1-(6-bromohexyl)-4-(4,5-dibromo-1-methyl-1H-pyrrol-2-yl)-1H-imidazo[4,5-c]pyridine (****3g****)**.* Yellow adhesive liquid (240 mg, yield 62%); ^1^H NMR (500 MHz, Chloroform-*d*) *δ* 8.42 (d, *J* = 5.6 Hz, 1H), 7.94 (s, 1H), 7.41 (s, 1H), 7.22 (d, *J* = 5.6 Hz, 1H), 4.20 (t, *J* = 7.1 Hz, 2H), 4.07 (s, 3H), 3.39 (t, *J* = 6.6 Hz, 2H), 1.92 (dt, *J* = 14.9, 7.3 Hz, 2H), 1.87 – 1.80 (m, 2H), 1.51 (dt, *J* = 15.2, 7.4 Hz, 2H), 1.36 (dt, *J* = 15.5, 7.7 Hz, 2H); ^13^C NMR (125 MHz, Chloroform-*d*) *δ* 143.75, 143.46, 141.02, 139.23, 137.79, 130.83, 116.80, 109.48, 103.48, 98.77, 45.12, 36.31, 33.49, 32.31, 29.73, 27.50, 25.89; HRMS (ESI^+^) *m/z*: Calcd for C_17_H_19_Br_3_N_4_; [M+H]^+^ 519.0790, Found 520.0890.

*1-(6-bromohexyl)-4-(4,5-dibromo-1H-pyrrol-2-yl)-1H-imidazo[4,5-c]pyridine -TFA (****4a****)**.* Treatments of **3c** (100 mg, 0.16 mmol) with BF_3_-OEt_2_ (0.2 mL, 1.6 mmol) and TFA (0.10 mL) carried out in the same manner as described for **1a** gave **4a.** Yellow amorphous solid (89 mg, 90%); ^1^H NMR (500 MHz, Methanol-*d*_4_) *δ* 8.66 (s, 1H), 8.30 (d, *J* = 6.5 Hz, 1H), 7.86 (d, *J* = 6.5 Hz, 1H), 7.43 (s, 1H), 4.43 (t, *J* = 7.3 Hz, 2H), 3.43 (t, *J* = 6.7 Hz, 2H), 1.97 (dt, *J* = 15.0, 7.5 Hz, 2H), 1.88 – 1.81 (m, 2H), 1.52 (dt, *J* = 14.9, 7.3 Hz, 2H), 1.41 (dt, *J* = 15.3, 7.5 Hz, 2H); ^13^C NMR (126 MHz, Methanol-*d*_4_) *δ* 148.79, 142.63, 135.89, 135.09, 133.86, 124.44, 116.82, 109.05, 105.43, 101.59, 45.47, 32.74, 32.24, 29.41, 27.19, 25.38; HRMS (ESI^+^) *m/z*: Calcd for C_16_H_18_Br_3_N_4_; [M+H]^+^ 504.9056, Found 504.9062.

*General Procedure for Compounds* ***5a*** *-* ***5c*** *and* ***7a****.* Compounds **3a, 3c,** and **3g** (0.16 mmol) were mixed with thiourea (1.6 mmol) or 1,1,3,3-tetramethylthiourea (1.6 mmol), respectively, in THF (20 mL) and refluxed overnight. The organic solvent was distilled off in vacuo and the reaction residue was subjected to silica gel column chromatography to obtain compounds **5a** - **5c** and **7a**.

*1-(4-(4-(4,5-dibromo-1-((2-(trimethylsilyl)ethoxy)methyl)-1H-pyrrol-2-yl)-1H-imidazo[4,5-c]pyridin-1-yl)butyl)isothiouronium hydrobromide (****5a****).* Yellow powder (45 mg, yield 47%); ^1^H NMR (500 MHz, DMSO-*d*_6_) *δ* 9.07 (s, 4H), 8.48 (s, 1H), 8.36 (d, *J* = 5.5 Hz, 1H), 7.66 (d, *J* = 5.6 Hz, 1H), 7.55 (s, 1H), 6.17 (s, 2H), 4.35 (t, *J* = 6.9 Hz, 2H), 3.27 (t, *J* = 8.1 Hz, 2H), 3.19 (t, *J* = 7.3 Hz, 2H), 1.90 (p, *J* = 7.1 Hz, 2H), 1.58 (p, *J* = 7.4 Hz, 2H), 0.61 (t, *J* = 8.1 Hz, 2H), -0.29 (s, 9H); ^13^C NMR (126 MHz, dmso) *δ* 170.12, 145.86, 142.27, 140.61, 139.77, 137.57, 131.86, 117.66, 109.53, 105.64, 100.01, 75.35, 65.15, 44.14, 29.88, 28.71, 26.20, 17.49, -1.22; HRMS (ESI^+^) *m/z*: Calcd for C_28_H_38_Br_2_N_6_O_4_S; [M+H]^+^ 603.0490, Found 603.0400.

*2-(6-(4-(4,5-dibromo-1-((2-(trimethylsilyl)ethoxy)methyl)-1H-pyrrol-2-yl)-1H-imidazo[4,5-c]pyridin-1-yl)hexyl)isothiouronium* *hydrobromide (****5b****).* Yellow powder (45 mg, yield 45%); ^1^H NMR (500 MHz, DMSO-*d*_6_) *δ* 9.08 (s, 4H), 8.47 (s, 1H), 8.35 (d, *J* = 5.5 Hz, 1H), 7.65 (d, *J* = 5.5 Hz, 1H), 7.54 (s, 1H), 6.17 (s, 2H), 4.30 (t, *J* = 6.9 Hz, 2H), 3.28 – 3.23 (m, 2H), 3.12 (t, *J* = 7.3 Hz, 2H), 1.79 (dt, *J* = 14.3, 7.0 Hz, 2H), 1.56 (dt, *J* = 14.7, 7.4 Hz, 2H), 1.42 – 1.35 (m, 2H), 1.29 – 1.23 (m, 2H), 0.63 – 0.58 (m, 2H), -0.30 (s, 9H); ^13^C NMR (125 MHz, DMSO-*d*_6_) *δ* 170.35, 145.91, 142.22, 140.54, 139.79, 137.56, 131.89, 117.61, 109.46, 105.62, 99.99, 75.31, 65.11, 44.71, 30.39, 29.66, 28.70, 27.70, 25.85, 17.46, -1.25; HRMS (ESI^+^) *m/z*: Calcd for C_23_H_35_Br_2_N_6_OSSi; [M+H]^+^ 631.5265, Found 631.0714.

*1-(6-(4-(4,5-dibromo-1-methyl-1H-pyrrol-2-yl)-1H-imidazo[4,5-c]pyridin-1-yl)hexyl)isothiouronium* *hydrobromide (****5c****).* Yellow powder (33 mg, yield 40%); ^1^H NMR (500 MHz, DMSO-*d_6_*) *δ* 9.06 (s, 4H), 8.44 (s, 1H), 8.34 (d, *J* = 5.5 Hz, 1H), 7.60 (d, *J* = 5.5 Hz, 1H), 7.52 (s, 1H), 4.28 (t, *J* = 6.8 Hz, 2H), 4.07 (s, 3H), 3.11 (t, *J* = 7.2 Hz, 2H), 1.82 – 1.75 (m, 2H), 1.55 (dt, *J* = 14.4, 7.3 Hz, 2H), 1.37 (dt, *J* = 14.7, 7.4 Hz, 2H), 1.27 – 1.22 (m, 2H); ^13^C NMR (125 MHz, DMSO-*d_6_*) *δ* 170.33, 145.75, 142.39, 140.64, 139.66, 137.37, 131.42, 116.62, 109.34, 105.34, 97.96, 44.71, 36.92, 30.38, 29.63, 28.68, 27.69, 25.86; HRMS (ESI^+^) *m/z*: Calcd for C_18_H_23_Br_2_N_6_S; [M+H]^+^ 515.0046, Found 515.0054.

*1-(6-(4-(4,5-dibromo-1-((2-(trimethylsilyl)ethoxy)methyl)-1H-pyrrol-2-yl)-1H-imidazo[4,5-c]pyridin-1-yl)hexyl)-1,1,3,3-tetramethylisothiouronium* *hydrobromide (****7a****).* Yellow powder (52 mg, yield 48%); ^1^H NMR (500 MHz, DMSO-*d*_6_) *δ* 8.48 (s, 1H), 8.35 (d, *J* = 5.5 Hz, 1H), 7.65 (d, *J* = 5.5 Hz, 1H), 7.55 (s, 1H), 6.18 (s, 2H), 4.31 (t, *J* = 6.9 Hz, 2H), 3.30 – 3.25 (m, 2H), 3.21 (s, 12H), 3.00 (t, *J* = 7.3 Hz, 2H), 1.80 (dd, *J* = 14.7, 7.2 Hz, 2H), 1.55 (dt, *J* = 14.8, 7.4 Hz, 2H), 1.38 (dt, *J* = 14.8, 7.6 Hz, 2H), 1.25 (dt, *J* = 15.2, 7.7 Hz, 2H), 0.65 – 0.59 (m, 2H), -0.29 (s, 9H); ^13^C NMR (125 MHz, DMSO-*d*_6_) *δ* 174.56, 145.94, 142.21, 140.53, 139.79, 137.54, 131.86, 117.63, 109.51, 105.66, 99.99, 75.34, 65.12, 44.72, 43.91, 34.16, 29.64, 29.51, 27.83, 25.91, 17.47, -1.23; HRMS (ESI^+^) *m/z*: Calcd for C_27_H_43_Br_2_N_6_OSSi; [M+H]^+^ 687.6345, Found 687.1333.

*General Procedure for Compounds* ***6a*** *-* ***6b*** *and* ***8a****.* HCl (1 mL) was added to a solution of **5a** - **5b** (0.05 mmol) in MeOH (20 mL) respectively, and the reaction was stirred at room temperature overnight. The solvent was evaporated under vacuum to get compounds **6a** - **6b** as brown amorphous solid.

*1-(4-(4-(4,5-dibromo-1H-pyrrol-2-yl)-1H-imidazo[4,5-c]pyridin-1-yl)butyl)isothiouronium hydrochloride hydrobromide (****6a****).* Yellow powder (27 mg, yield 92%); ^1^H NMR (500 MHz, DMSO-*d_6_*) *δ* 13.57 (s, 1H), 9.34 (s, 2H), 8.94 (s, 1H), 8.43 (d, *J* = 5.6 Hz, 1H), 8.10 (d, *J* = 5.0 Hz, 1H), 7.96 (s, 1H), 4.49 (s, 2H), 3.28 (t, *J* = 6.6 Hz, 2H), 2.02 – 1.89 (m, 2H), 1.62 (s, 2H); ^13^C NMR (125 MHz, DMSO-*d_6_*) *δ* 170.33, 149.76, 142.77, 134.92, 106.71, 101.28, 44.93, 29.81, 28.68, 26.18; HRMS (ESI^+^) *m/z*: Calcd for C_15_H_17_Br_2_N_6_S; [M+H^+^] 472.9576, Found 472.9571.

*1-(6-(4-(4,5-dibromo-1H-pyrrol-2-yl)-1H-imidazo[4,5-c]pyridin-1-yl)hexyl)isothiouronium hydrochloride hydrobromide (****6b****).* Yellow powder (28 mg, yield 91%); ^1^H NMR (400 MHz, Methanol-*d*_4_) *δ* 8.81 (s, 1H), 8.35 (d, *J* = 6.7 Hz, 1H), 8.04 (d, *J* = 6.7 Hz, 1H), 7.60 (s, 1H), 4.52 (t, *J* = 7.2 Hz, 2H), 3.18 (t, *J* = 7.3 Hz, 2H), 2.06 – 1.96 (m, 2H), 1.80 – 1.70 (m, 2H), 1.61 – 1.52 (m, 2H), 1.47 (dt, *J* = 14.5, 7.5 Hz, 2H); ^13^C NMR (100 MHz, Methanol-*d*_4_) *δ* 171.62, 149.72, 144.46, 143.36, 135.01, 134.88, 132.37, 123.04, 117.81, 117.49, 110.38, 105.92, 105.87, 102.07, 30.31, 29.43, 28.12, 27.55, 25.69; HRMS (ESI^+^) *m/z*: Calcd for C_17_H_21_Br_2_N_6_S; [M+H]^+^ 500.9889, Found 500.9901.

*1-(6-(4-(4,5-dibromo-1H-pyrrol-2-yl)-1H-imidazo[4,5-c]pyridin-1-yl)hexyl)-1,1,3,3-tetramethylisothiouronium hydrochloride hydrobromide (****8a****).* Yellow powder (27 mg, yield 80%); ^1^H NMR (400 MHz, Methanol-*d*_4_) *δ* 8.82 (s, 1H), 8.36 (s, 1H), 8.06 (s, 1H), 7.59 (s, 1H), 4.53 (s, 2H), 3.37 (s, 3H), 3.10 (s, 2H), 2.03 (s, 2H), 1.75 (s, 2H), 1.53 (d, *J* = 28.7 Hz, 4H); ^13^C NMR (100 MHz, Methanol-*d*_4_) *δ* 176.00, 149.90, 143.44, 135.10, 134.97, 132.53, 123.08, 117.78, 110.45, 106.15, 102.11, 45.83, 43.15, 34.30, 29.55, 29.31, 27.87, 25.89; HRMS (ESI^+^) *m/z*: Calcd for C_21_H_30_Br_2_N_6_S; [M+H]^+^ 557.0515, Found 557.0508.

*General Procedure for Compounds* ***9a*** *-* ***9d****.* To a stirred solution of **3c** (0.16 mmol) in DMF (30 mL) was added K_2_CO_3_ (0.32 mmol) and appropriate heterocycle (0.32 mmol) at room temperature for 3 h. The reaction mixture was extracted with EtOAc. The combined extracts were washed with H_2_O and brine, dried over anhydrous MgSO_4_, filtered, and then concentrated in vacuo. The reaction residue was subjected to preliminary purification on a silica gel column (petroleum ether/ethyl acetate 25:1) to obtain **9a** - **9d**.

*1-(6-(4-(4,5-dibromo-1-((2-(trimethylsilyl)ethoxy)methyl)-1H-pyrrol-2-yl)-1H-imidazo[4,5-c]pyridin-1-yl)hexyl)morpholine (****9a****).* Yellow adhesive liquid (81 mg, yield 80%); ^1^H NMR (500 MHz, Chloroform-*d*) *δ* 8.40 (d, *J* = 5.5 Hz, 1H), 7.94 (s, 1H), 7.51 (s, 1H), 7.22 (d, *J* = 5.5 Hz, 1H), 6.18 (s, 3H), 4.18 (t, *J* = 7.1 Hz, 3H), 3.70 (t, *J* = 4.5 Hz, 4H), 3.49 – 3.28 (m, 2H), 2.41 (s, 4H), 2.32 – 2.27 (m, 2H), 1.93 – 1.86 (m, 2H), 1.52 – 1.44 (m, 2H), 1.36 (s, 4H), 0.75 – 0.69 (m, 2H), -0.23 (s, 9H); ^13^C NMR (125 MHz, Chloroform-*d*) *δ* 143.62, 143.58, 140.98, 139.43, 137.85, 131.31, 118.21, 103.77, 75.46, 67.01, 65.51, 58.89, 53.83, 45.31, 29.94, 26.99, 26.77, 26.41, 17.72, -1.56; HRMS (ESI^+^) *m/z*: Calcd for C_26_H_39_Br_2_N_5_O_2_Si; [M+H]^+^ 641.5240, Found 642.1295.

*1-(6-(4,5-dibromo-1-((2-(trimethylsilyl)ethoxy)methyl)-1H-pyrrol-2-yl)-1-(6-(4-methylpiperazin-1-yl)hexyl)-1H-imidazo[4,5-c]pyridine (****9b****).* Yellow adhesive liquid (84 mg, yield 80%); ^1^H NMR (500 MHz, Chloroform-*d*) *δ* 8.40 (d, *J* = 5.5 Hz, 1H), 7.94 (s, 1H), 7.51 (s, 1H), 7.22 (d, *J* = 5.6 Hz, 1H), 6.18 (s, 2H), 4.18 (t, *J* = 7.1 Hz, 2H), 3.40 – 3.31 (m, 2H), 2.47 (s, 4H), 2.34 – 2.30 (m, 2H), 2.29 (s, 3H), 1.92 (d, *J* = 29.2 Hz, 6H), 1.48 (s, 2H), 1.36 (s, 4H), 0.75 – 0.69 (m, 2H), -0.23 (s, 9H); ^13^C NMR (126 MHz, Chloroform-*d*) *δ* 143.50, 140.88, 139.33, 137.74, 135.59, 131.21, 118.06, 109.44, 103.71, 100.77, 75.34, 65.41, 58.32, 54.92, 53.04, 45.90, 45.22, 29.83, 26.95, 26.66, 26.58, 17.61, -1.65; HRMS (ESI^+^) *m/z*: Calcd for C_27_H_42_Br_2_N_6_OSi; [M+H]^+^ 654.5670, Found 655.1613.

*1-(6-(4-(4,5-dibromo-1-((2-(trimethylsilyl)ethoxy)methyl)-1H-pyrrol-2-yl)-1H-imidazo[4,5-c]pyridin-1-yl)hexyl)isoindoline-1,3-dione (****9c****).* White solid (95 mg, yield 85%); ^1^H NMR (500 MHz, Chloroform-*d*) *δ* 8.39 (d, *J* = 5.6 Hz, 1H), 7.95 (s, 1H), 7.84 (q, *J* = 3.0 Hz, 2H), 7.71 (dd, *J* = 5.4, 3.1 Hz, 2H), 7.50 (s, 1H), 7.22 (d, *J* = 5.6 Hz, 1H), 6.17 (s, 2H), 4.17 (t, *J* = 7.2 Hz, 2H), 3.68 (t, *J* = 7.1 Hz, 2H), 3.36 (t, *J* = 8.3 Hz, 2H), 1.93 – 1.86 (m, 2H), 1.72 – 1.65 (m, 2H), 1.41 (dt, *J* = 7.1, 3.4 Hz, 4H), 0.71 (t, *J* = 8.3 Hz, 2H), -0.23 (s, 9H); ^13^C NMR (125 MHz, Chloroform-*d*) *δ* 168.41, 143.48, 140.89, 139.32, 137.76, 133.96, 132.04, 131.23, 123.21, 118.06, 109.41, 103.70, 100.78, 65.40, 45.19, 37.58, 29.75, 29.68, 28.29, 26.23, 26.20, 17.62, -1.66; HRMS (ESI^+^) *m/z*: Calcd for C_30_H_35_Br_2_N_5_O_3_Si; [M+H]^+^ 654.5670, Found 701.5350.

*1-(6-(4,5-dibromo-1-((2-(trimethylsilyl)ethoxy)methyl)-1H-pyrrol-2-yl)-1-(6-(piperidin-1-yl)hexyl)-1H-imidazo[4,5-c]pyridine (****9d****).* Yellow adhesive liquid (81 mg, yield 79%); ^1^H NMR (500 MHz, Methanol-*d*_4_) *δ* 8.42 (d, *J* = 5.6 Hz, 1H), 8.35 (s, 1H), 7.63 (d, *J* = 5.6 Hz, 1H), 7.14 (s, 1H), 5.98 (s, 2H), 4.37 (t, *J* = 7.0 Hz, 2H), 3.23 (t, *J* = 8.0 Hz, 2H), 3.12 (s, 2H), 3.00 – 2.94 (m, 2H), 1.98 – 1.92 (m, 2H), 1.82 (s, 4H), 1.74 – 1.62 (m, 4H), 1.42 (s, 4H), 0.61 (t, *J* = 8.0 Hz, 2H), -0.25 (s, 9H); ^13^C NMR (125 MHz, Methanol-*d*_4_) *δ* 145.53, 142.59, 140.68, 139.84, 137.49, 131.34, 116.73, 108.45, 105.07, 100.04, 75.16, 65.20, 56.87, 52.96, 44.72, 29.29, 25.84, 25.82, 23.68, 23.07, 21.53, 16.95, -2.97; HRMS (ESI^+^) *m/z*: Calcd for C_27_H_41_Br_2_N_5_OSi; [M+H]^+^ 639.5520, Found 640.1497.

*General Procedure for Compounds* ***10a*** *-* ***10d****.* Similar treatments of **9a** - **9d** (0.12 mmol) with BF_3_-OEt_2_ (1.2 mmol) and TFA (0.10 mL) to those described for **2a** gave **10a** - **10d** as a pale yellow powder after trituration with CH_2_Cl_2_-MeOH.

*1-(6-(4-(4,5-dibromo-1H-pyrrol-2-yl)-1H-imidazo[4,5-c]pyridin-1-yl)hexyl)morpholine -TFA (****10a****).* Yellow powder (66 mg, yield 85%); ^1^H NMR (500 MHz, Methanol-*d*_4_) *δ* 8.67 (s, 1H), 8.32 (d, *J* = 6.5 Hz, 1H), 7.88 (d, *J* = 6.5 Hz, 1H), 7.49 (s, 1H), 4.45 (t, *J* = 7.2 Hz, 2H), 4.04 (d, *J* = 12.0 Hz, 2H), 3.75 (t, *J* = 11.5 Hz, 2H), 3.47 (d, *J* = 12.3 Hz, 2H), 3.15 – 3.06 (m, 4H), 2.02 – 1.94 (m, 2H), 1.75 (dt, *J* = 15.6, 7.7 Hz, 2H), 1.48 – 1.42 (m, 4H); ^13^C NMR (125 MHz, Methanol-*d*_4_) *δ* 149.43, 143.93, 143.17, 135.35, 135.12, 132.92, 123.49, 117.42, 105.75, 101.98, 56.66, 52.87, 45.50, 29.32, 25.70, 25.67, 23.44, 22.87, 21.31; HRMS (ESI^+^) m/z: Calcd for C_20_H_26_Br_2_N_5_O; [M+H]^+^ 512.0478, Found 512.0482.

*1-(6-(4,5-dibromo-1H-pyrrol-2-yl)-1-(6-(4-methylpiperazin-1-yl)hexyl)-1H-imidazo[4,5-c]pyridine -TFA (****10b****).* Yellow powder (65 mg, yield 85%); ^1^H NMR (500 MHz, Methanol-*d*_4_) *δ* 8.81 (s, 1H), 8.34 (d, *J* = 6.6 Hz, 1H), 8.04 (d, *J* = 6.6 Hz, 1H), 7.63 (s, 1H), 4.50 (t, *J* = 7.1 Hz, 2H), 3.72 (s, 8H), 3.30 – 3.25 (m, 3H), 3.01 (s, 3H), 2.04 – 1.98 (m, 2H), 1.87 – 1.79 (m, 2H), 1.54 – 1.45 (m, 4H); ^13^C ^13^C NMR (500 MHz, Methanol-*d*_4_) *δ* 160.82, 160.53, 149.67, 143.29, 134.99, 132.42, 123.11, 117.97, 115.11, 110.21, 105.96, 101.98, 56.22, 49.84, 45.55, 41.95, 29.19, 25.47, 25.41, 23.19; HRMS (ESI^+^) *m/z*: Calcd for C_21_H_29_Br_2_N_6_; [M+H]^+^ 525.0794, Found 525.0800.

*1-(6-(4-(4,5-dibromo-1H-pyrrol-2-yl)-1H-imidazo[4,5-c]pyridin-1-yl)hexyl)isoindoline-1,3-dione -TFA (****10c****).* Yellow powder (63 mg, yield 83%); ^1^H NMR (500 MHz, Methanol-*d*_4_) *δ* 8.69 (s, 1H), 8.31 (d, *J* = 6.4 Hz, 1H), 7.90 (d, *J* = 6.3 Hz, 1H), 7.82 – 7.75 (m, 4H), 7.46 (s, 1H), 4.44 (t, *J* = 7.1 Hz, 2H), 3.63 (t, *J* = 7.0 Hz, 2H), 2.00 – 1.92 (m, 2H), 1.70 – 1.63 (m, 2H), 1.47 – 1.36 (m, 4H); ^13^C NMR (125 MHz, Methanol-*d*_4_) *δ* 168.41, 149.12, 142.91, 135.13, 133.92, 133.33, 131.86, 123.98, 122.60, 116.97, 105.57, 101.79, 45.55, 37.05, 29.31, 27.76, 25.81, 25.63; HRMS (ESI^+^) *m/z*: Calcd for C_24_H_22_Br_2_N_5_O_2_; [M+H]^+^ 572.0114, Found 572.0109.

*1-(6-(4,5-dibromo-1H-pyrrol-2-yl)-1-(6-(piperidin-1-yl)hexyl)-1H-imidazo[4,5-c]pyridine -TFA (****10d****).* Yellow powder (68 mg, yield 85%); ^1^H NMR (400 MHz, Methanol-*d*_4_) *δ* 8.76 (s, 1H), 8.35 (d, *J* = 6.5 Hz, 1H), 7.98 (d, *J* = 6.5 Hz, 1H), 7.54 (s, 1H), 4.49 (t, *J* = 7.2 Hz, 2H), 3.53 (d, *J* = 12.4 Hz, 2H), 3.12 – 3.04 (m, 2H), 2.91 (t, *J* = 12.4 Hz, 2H), 2.10 – 1.68 (m, 12H); ^13^C NMR (100 MHz, Methanol-*d*_4_) *δ* 149.43, 143.93, 143.17, 135.35, 135.12, 132.92, 123.49, 117.42, 110.06, 109.05, 105.75, 101.98, 56.66, 52.87, 45.50, 29.32, 25.70, 25.67, 23.44, 22.87, 21.31; HRMS (ESI^+^) *m/z*: Calcd for C_21_H_28_Br_2_N_5_; [M+H]^+^ 510.0686, Found 510.0686.

*General Procedure for Compounds* ***11a*** *-* ***11c****.*

*1-(6-bromohexyl)-4-(4,5-dibromo-1-(6-bromohexyl)-1H-pyrrol-2-yl)-1H-imidazo[4,5-c]pyridine (****11a****)**.* To a stirred solution of **4a** (500 mg, 1.1 mmol) in DMF (30 mL) was added Cs_2_CO_3_ (1.43 g, 4.38 mmol) and 1,6-dibromohexane (0.67 mL, 4.38 mmol) at room temperature for 3 h. The reaction mixture was extracted with EtOAc. The combined extracts were washed with H_2_O and brine, dried over anhydrous MgSO_4_, filtered, and then concentrated in vacuo. The reaction residue was subjected to preliminary purification on a silica gel column (petroleum ether/ethyl acetate 10:1) to obtain **10a** (367 mg, 50%). ^1^H NMR (600 MHz, Chloroform-*d*) *δ* 8.39 (d, *J* = 5.6 Hz, 1H), 7.94 (s, 1H), 7.51 (s, 1H), 7.21 (d, *J* = 5.6 Hz, 1H), 4.72 – 4.64 (m, 2H), 4.19 (t, *J* = 7.1 Hz, 2H), 3.38 (t, *J* = 6.6 Hz, 2H), 3.30 (t, *J* = 6.9 Hz, 2H), 1.92 (dt, *J* = 14.9, 7.3 Hz, 2H), 1.86 – 1.81 (m, 2H), 1.74 (dd, *J* = 14.5, 7.3 Hz, 2H), 1.69 (dd, *J* = 14.1, 6.6 Hz, 2H), 1.66 – 1.60 (m, 2H), 1.50 (dt, *J* = 15.3, 7.5 Hz, 2H), 1.39 – 1.33 (m, 4H); ^13^C NMR (150 MHz, Chloroform-*d*) *δ* 143.78, 143.49, 141.00, 139.37, 137.66, 130.21, 117.49, 108.82, 103.54, 99.24, 48.13, 45.23, 33.85, 33.55, 32.63, 32.40, 30.42, 29.83, 27.67, 27.60, 26.00, 25.59; HRMS (ESI^+^) *m/z*: Calcd for C_22_H_31_Br_2_N_6_S; [M+H]^+^ 668.1100, Found 669.1000.

*1-(6-(4-(4,5-dibromo-1-(6-morpholinohexyl)-1H-pyrrol-2-yl)-1H-imidazo[4,5-c]pyridin-1-yl)hexyl)morpholine (****11b****).* The same treatments of **11a** (100 mg, 0.15 mmol) with K_2_CO_3_ ((84 mg, 0.6 mmol) and morpholine (53 mg, 0.6 mmol) as those described for **9a** gave **11b** (84 mg, 83%) as a yellow adhesive liquid. ^1^H NMR (500 MHz, Chloroform-*d*) *δ* 8.38 (d, *J* = 5.5 Hz, 1H), 7.93 (s, 1H), 7.54 (s, 1H), 7.20 (d, *J* = 5.5 Hz, 1H), 4.74 – 4.64 (m, 2H), 4.18 (t, *J* = 7.1 Hz, 2H), 3.71 (s, 8H), 2.43 (s, 8H), 2.34 – 2.25 (m, 4H), 1.94 – 1.86 (m, 2H), 1.73 – 1.66 (m, 2H), 1.52 – 1.46 (m, 2H), 1.45 – 1.40 (m, 2H), 1.37 (s, 4H), 1.25 (s, 4H); ^13^C NMR (125 MHz, Chloroform-*d*) *δ* 143.73, 143.31, 140.89, 139.21, 137.51, 130.18, 117.34, 108.69, 103.37, 99.03, 66.83, 66.75, 58.95, 58.77, 53.69, 53.62, 48.23, 45.21, 30.57, 29.83, 26.93, 26.88, 26.66, 26.29, 26.22, 26.08; HRMS (ESI^+^) *m/z*: Calcd for C_30_H_45_Br_2_N_6_O_2_; [M+H]^+^ 681.1950, Found 681.1949.

*1-(6-(4-(1-(6-((amino(iminio)methyl)thio)hexyl)-4,5-dibromo-1H-pyrrol-2-yl)-1H-imidazo[4,5-c]pyridin-1-yl)hexyl)isothiouronium hydrobromide (****11c****).* Treatments of **11a** (100 mg, 0.15 mmol) with thiourea (103 mg, 1.35 mmol) carried out in the same manner as described for **5a** gave **11c** (55 mg, 45%) as a brown adhesive liquid. ^1^H NMR (400 MHz, Chloroform-*d*) *δ* 8.43 (d, *J* = 5.6 Hz, 1H), 8.40 (s, 1H), 7.67 (d, *J* = 5.6 Hz, 1H), 7.10 (s, 1H), 4.59 (t, *J* = 7.0 Hz, 2H), 4.41 (t, *J* = 7.1 Hz, 2H), 3.17 (t, *J* = 7.3 Hz, 2H), 3.03 (t, *J* = 7.3 Hz, 2H), 1.97 (dt, *J* = 14.5, 7.1 Hz, 2H), 1.74 (dt, *J* = 14.8, 7.4 Hz, 2H), 1.52 (m, 10H), 1.17 (dd, *J* = 14.6, 7.6 Hz, 2H); ^13^C NMR (100 MHz, Chloroform-*d*) *δ* 171.61, 145.84, 142.53, 140.52, 139.97, 137.42, 130.21, 116.06, 107.64, 105.38, 98.53, 47.36, 44.99, 30.46, 30.33, 29.92, 29.38, 28.15, 27.86, 27.52, 27.29, 25.72, 25.07; HRMS (ESI^+^) *m/z*: Calcd for C_24_H_35_Br_2_N_8_S_2_; [M+H]^+^ 659.0772, Found 659.0764.

**Scheme 1.** Synthesis of Compounds **1c, 2a, 2b** and **4a**

Reagents and conditions:(a) NBS, THF, 0 ℃ ~ rt, 3 h; (b) SEMCl, *t*-BuOK, rt, overnight; (c) CH_3_I, K_2_CO_3_, DMF, rt, overnight; (d) Histamine dihydrochloride, EtOH, 80 ℃, 12 h; (e) IBX, DMSO, rt, 2 h; MnO_2_, DCM, rt, 2.5 h; (f) Boron (tri) fluoride etherate, DCM, 15 h, trifluoroacetic acid; (g) Cs_2_CO_3_, DMF, Br (CH_2_)_n_Br, rt, 3 h.

Condensation reactions of commercially available histamine dihydrochloride with aldehyde derivatives 3 - 5 in EtOH was carried out in the presence of an appropriate amount of triethylamine at 80 ℃ for 12 h to generated 6 - 8 as an intermediate with yields between 40% to 60%. And the derivatives 1a - 1c was produced in a two-step dehydrogenation by treating 3 - 5 with 1.5 equiv IBX and activated MnO_2_. Notably, in order to ensure the high activity of MnO_2_, it is necessary to make it on the spot. The derivatives 1a and 1b synthesized above were deprotected by BF_3_-OEt_2_ and trifluoroacetic acid (TFA) to afford 2a and 2b in high yields. The intermediates 3a - 3g were obtained by introducing different length of brominated alkly linker from 1a - 1c, with yields at 45% to 60%. Deprotection of 3c in the same manner as that for the preparation of 2a give 4a with a yield of 90%.

**Scheme 2.** Synthesis of Compounds **5c, 6a, 6b** and **8a**

Reagents and conditions:(a) Thiourea or 1,1,3,3-Tetramethylthiourea, THF, 70 ℃, overnight; (b) HCl, MeOH, 70 ℃, overnight.

To explore the effect of introducing isothiourea or tetramethyl-isothiourea group through different lengths of alkly chains, we synthesized four new Ageladine A derivatives. Scheme 2 illustrates the synthetic routes of compounds **5c**, **6a**, **6b** and **8a**. The intermediates **5a** - **5c** and **7a** could be prepared by refluxing **3a**, **3c** and **3g** with thiourea or 1,1,3,3-tetramethylthiourea in THF for a long time with yields at 40% to 50%. The compounds **6a**, **6b** and **8a** were obtained from **5a**, **5b** and **7a** by removing the -SEM under the HCl in MeOH with yields at 75% or so. This is different from the previously mentioned deprotection method, because these compounds are more water-soluble, causing problems in the extraction process.

**Scheme 3.** Synthesis of Compounds **10a** - **10d**

Reagents and conditions:(a) Heterocycle, K_2_CO_3,_ DMF, rt, overnight; (b)Boron (tri) fluoride etherate, DCM, 15 h, trifluoroacetic acid.

To explore the effect of the substituent with heteroring group at NH of imidazole, or with the same substituent at the NH of pyrrole and imidazole, we synthesized four Ageladine A derivatives with different heterocycle group and three Ageladine A derivatives with two identical substituents. Scheme 3 and Scheme 4 illustrates the synthetic routes of compounds **10a** - **10d** and **11a** - **11c**.

In the presence of K_2_CO_3_, **3c** were treated with different types of heterocyclic, such as piperidine, furan, etc to generate **9a** - **9d** at yields about 80%. And the compounds **10a** - **10d** were obtained after deprotection shown in Scheme 1 with high yields. Simultaneous insertion of bromine linker on imidazole and pyrrole rings of compound 2a affords 11a at a yield of 50%. 11b and 10c were prepared according to the similar means as shown in Scheme 2 and Scheme 3, with yields of 83%, 43%, respectively.

**Scheme 4.** Synthesis of Compounds **11a** - **11c**

Reagents and conditions: (a) Cs_2_CO_3_, DMF, Br(CH_2_)_6_Br, rt, overnight; (b) Morpholine, K_2_CO_3,_ DMF, rt, overnight; (c) Thiourea, THF, 70 ℃, overnight.

^1^H NMR Spectrum of **1c** in Methanol-*d*_4_

^13^C NMR Spectrum of **1c** in Methanol-*d*_4_

^1^H NMR Spectrum of **2a** in DMSO-*d*_6_

^13^C NMR Spectrum of **2a** in DMSO-*d*_6_

^1^H NMR Spectrum of **4a** in DMSO-*d*_6_

^13^C NMR Spectrum of **4a** in DMSO-*d*_6_

^1^H NMR Spectrum of **5c** in DMSO-*d*_6_

^13^C NMR Spectrum of **5c** in DMSO-*d*_6_

^1^H NMR Spectrum of **6a** in DMSO-*d*_6_

^13^C NMR Spectrum of **6a** in DMSO-*d*_6_

^1^H NMR Spectrum of **6b** in Methanol-*d*_4_

^13^C NMR Spectrum of **6b** in Methanol-*d*_4_

^1^H NMR Spectrum of **8a** in Methanol-*d*_4_

^13^C NMR Spectrum of **8a** in Methanol-*d*_4_

^1^H NMR Spectrum of **10a** in Methanol-*d*_4_

^13^C NMR Spectrum of **10a** in Methanol-*d*_4_

^1^H NMR Spectrum of **10b** in Methanol-*d*_4_

^13^C NMR Spectrum of **10b** in Methanol-*d*_4_

^1^H NMR Spectrum of **10c** in Methanol-*d*_4_

^13^C NMR Spectrum of **10c** in Methanol-*d*_4_

^1^H NMR Spectrum of **10d** in Methanol-*d*_4_

^13^C NMR Spectrum of **10d** in Methanol-*d*_4_

^1^H NMR Spectrum of **11a** in Chloroform-*d*

^13^C NMR Spectrum of **11a** in Chloroform-*d*

^1^H NMR Spectrum of **11b** in Chloroform-*d*

^13^C NMR Spectrum of **11b** in Chloroform-*d*

^1^H NMR Spectrum of **11c** inMethanol-*d*_4_

^13^C NMR Spectrum of **11c** inMethanol-*d*_4_

HRMS (ESI) spectra of compound **5c**


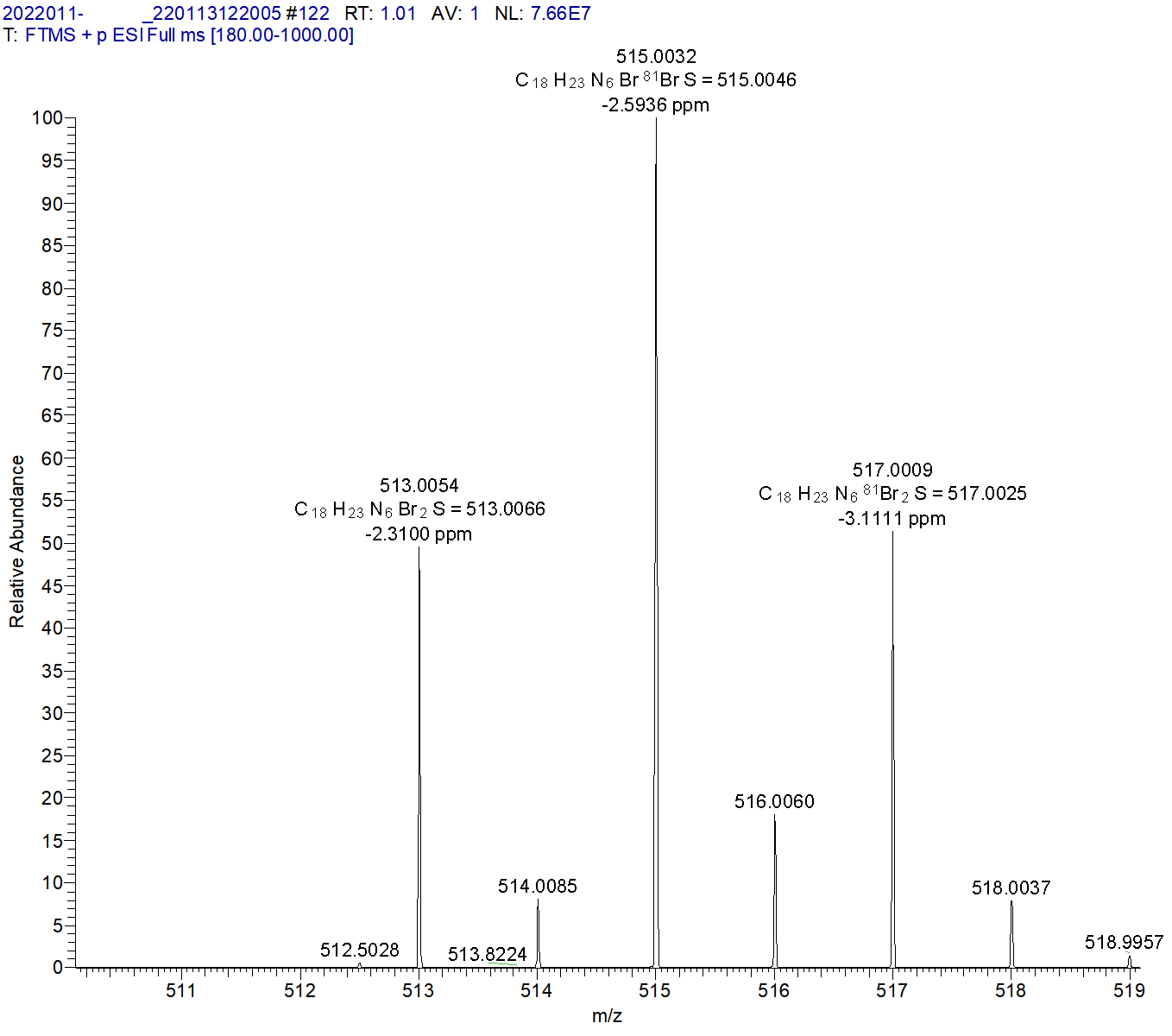


HRMS (ESI) spectra of compound **6b**


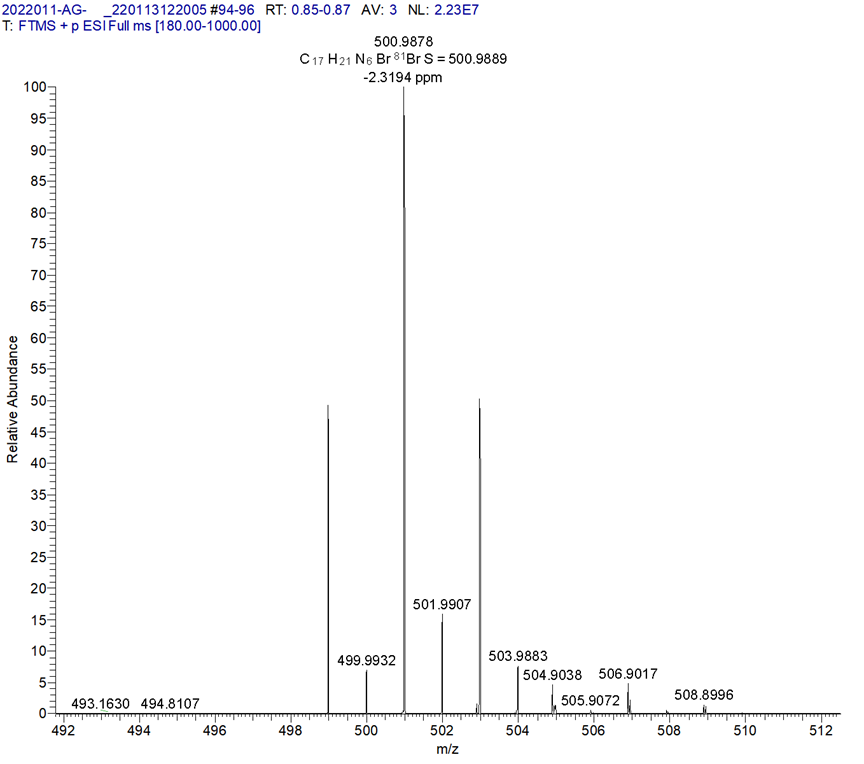


HRMS (ESI) spectra of compound **8a**


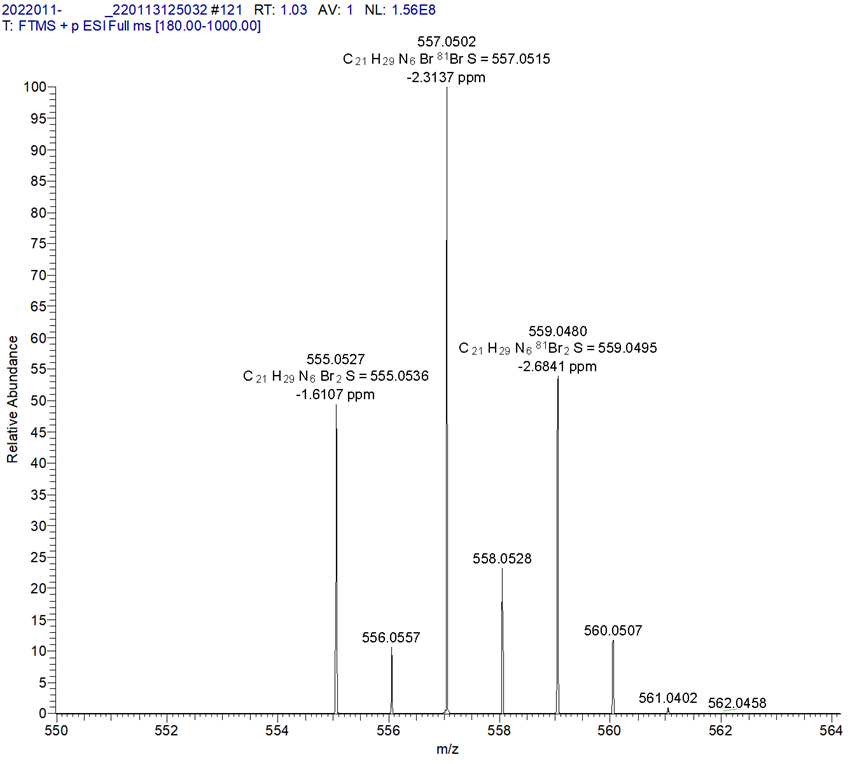

Supplement: Supplementary file 1 — Supplementary information [file 41419_2023_6018_MOESM1_ESM.docx]
