# Supplementary material for: Discovery of a pyrrole-pyridinimidazole derivative as novel SIRT6 inhibitor for sensitizing pancreatic cancer to gemcitabine
Source: Cell Death Dis. 2023 Aug 4;14(8):499. doi: 10.1038/s41419-023-06018-1 (PMC10403574; doi:10.1038/s41419-023-06018-1)
Supplement: Supplementary file 2 — Western Blot [file 41419_2023_6018_MOESM2_ESM.pdf]

1. Figure 1

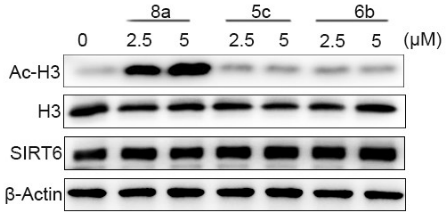
8a
5c
6b
0
2.5
5
2.5
5
2.5
5
(μM)
Ac-H3
H3
SIRT6
β-Actin

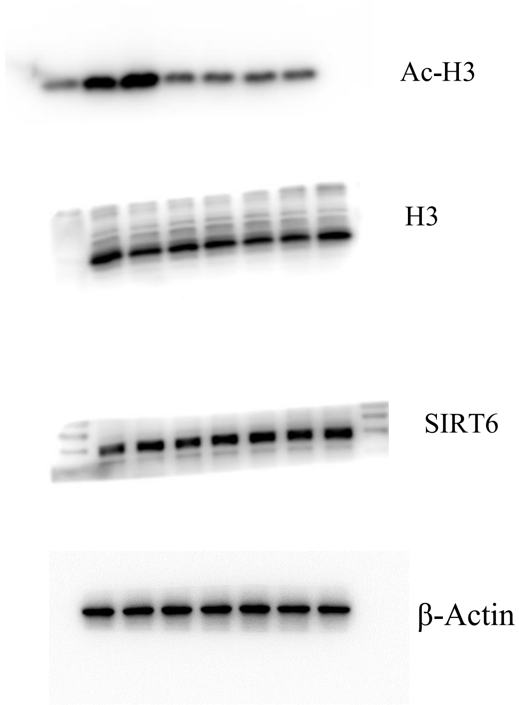
Ac-H3
H3
SIRT6
β-Actin

2．Figure 2

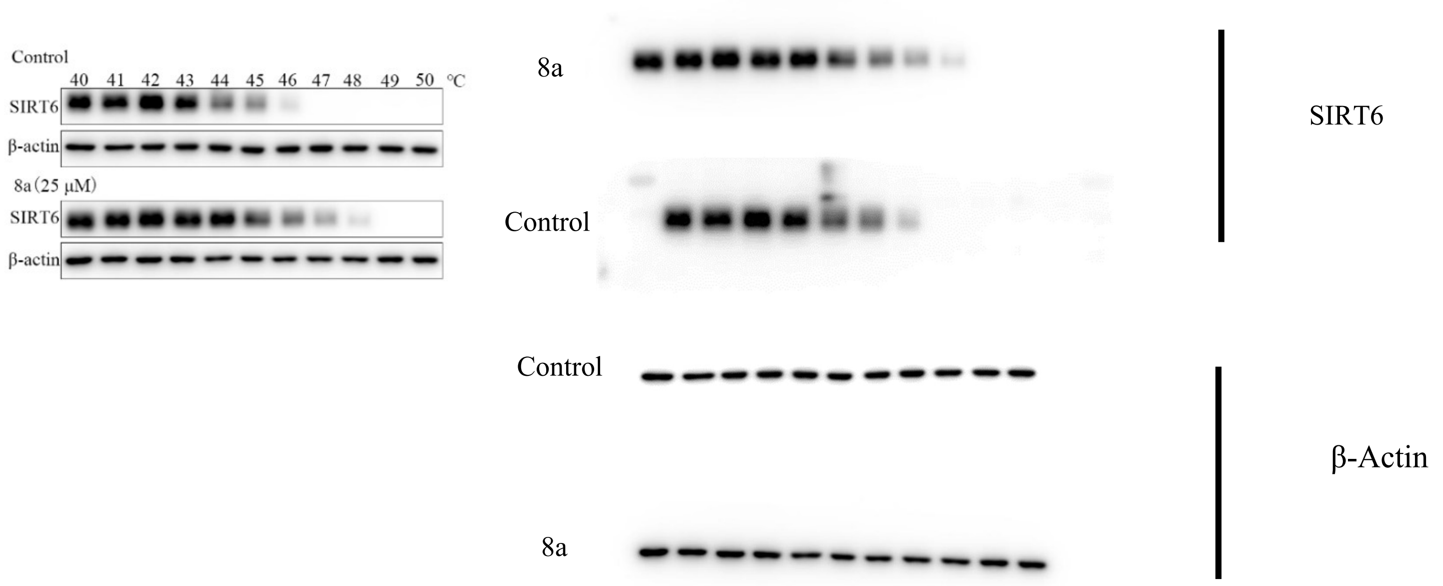

Control
40 41 42 43 44 45 46 47 48 49 50 °C
SIRT6
β-actin
8a (25 μM)
SIRT6
β-actin
8a
Control
SIRT6
Control
8a
β-Actin

3. Figure 3a

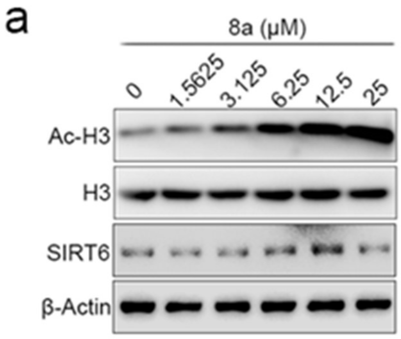
a
8a (μM)
0
1.5625
3.125
6.25
12.5
25
Ac-H3
H3
SIRT6
β-Actin

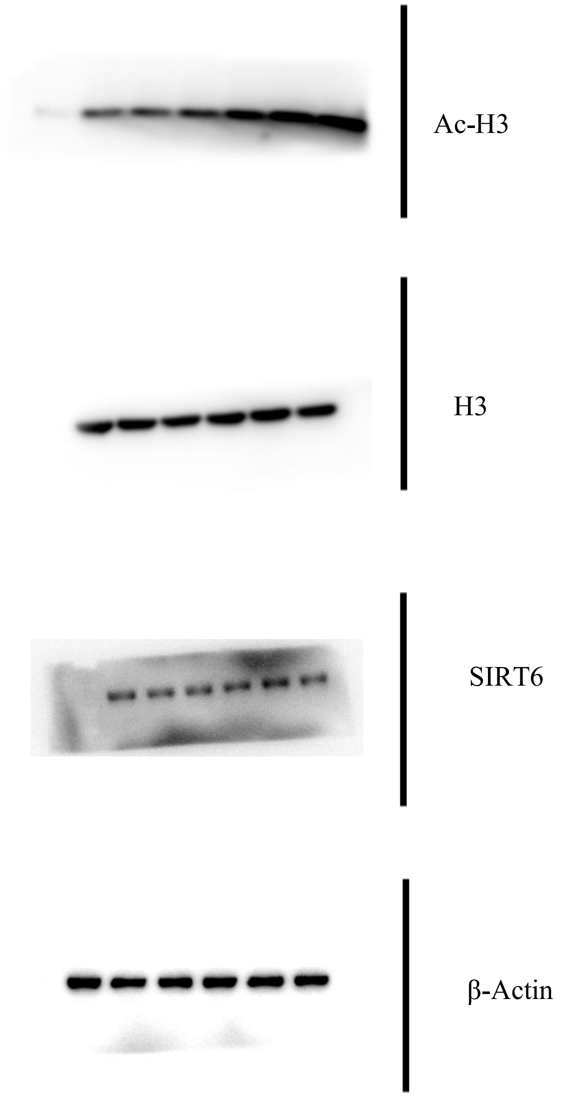
Ac-H3
H3
SIRT6
β-Actin

4. Figure 3d

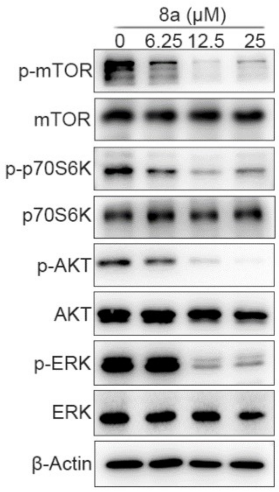
8a (μM)
0
6.25
12.5
25
p-mTOR
mTOR
p-p70S6K
p70S6K
p-AKT
AKT
p-ERK
ERK
β-Actin

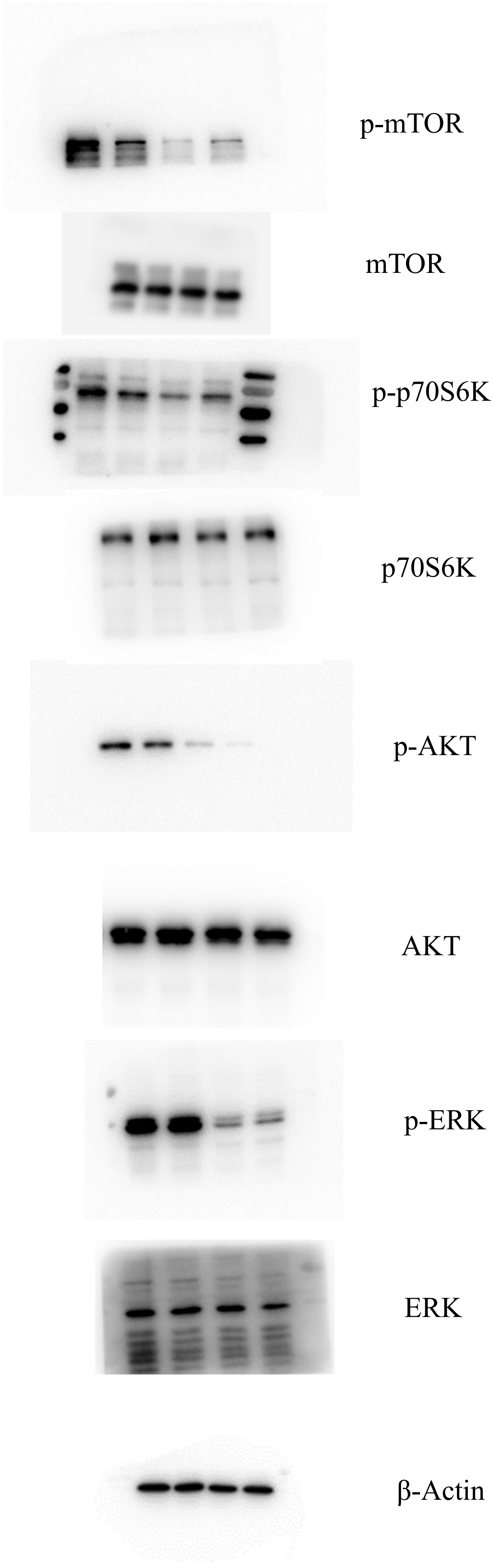
p-mTOR
mTOR
p-p70S6K
p70S6K
p-AKT
AKT
p-ERK
ERK
β-Actin

5. Figure 3g

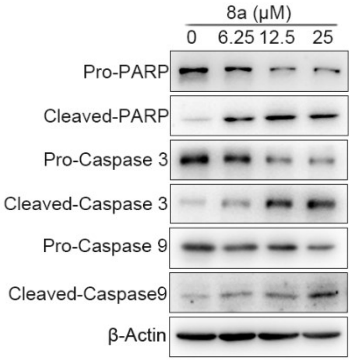

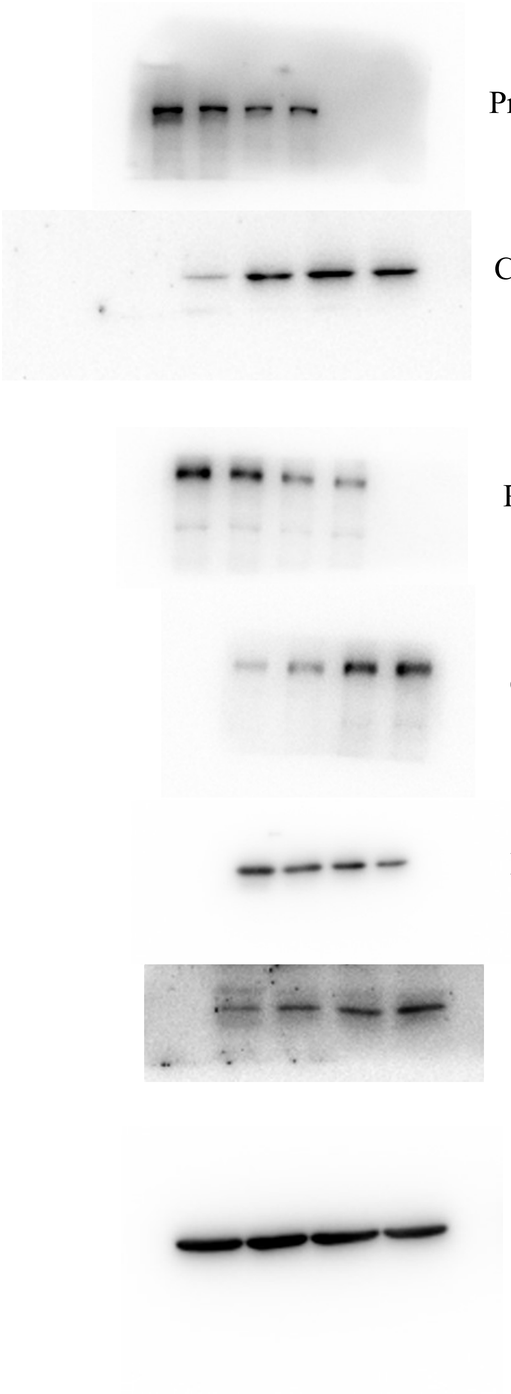

Pro-PARP

Cleaved-PARP

Pro-Caspase 3

Cleaved-Caspase 3

Pro-Caspase 9

Cleaved-Caspase 9

β-Actin

6. Figure 4e

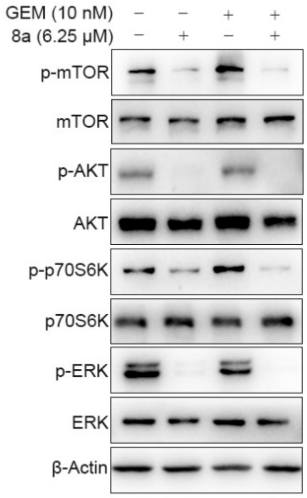
GEM (10 nM) − − + +
8a (6.25 μM) − + − +
p-mTOR
mTOR
p-AKT
AKT
p-p70S6K
p70S6K
p-ERK
ERK
β-Actin

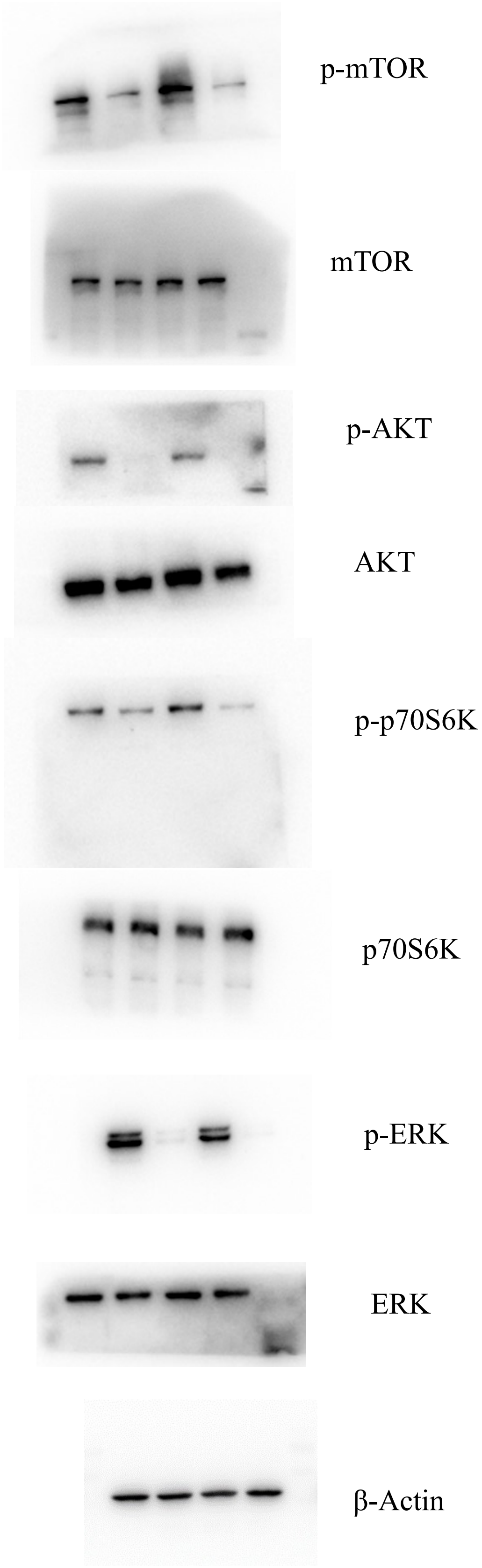
p-mTOR
mTOR
p-AKT
AKT
p-p70S6K
p70S6K
p-ERK
ERK
β-Actin

7. Figure 4g

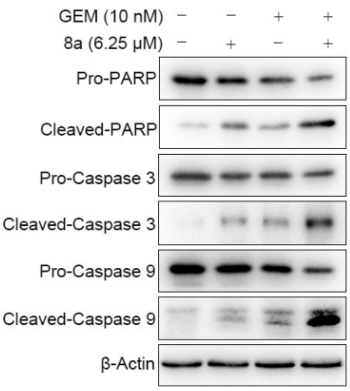

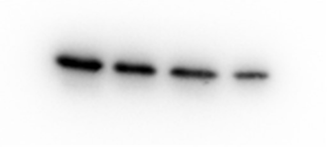

Pro-PARP

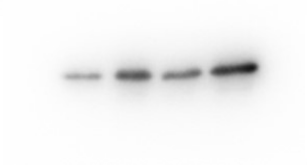

Cleaved-PARP

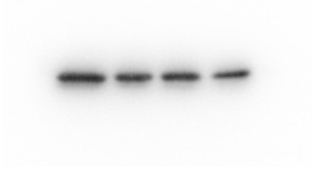

Pro-Caspase 3

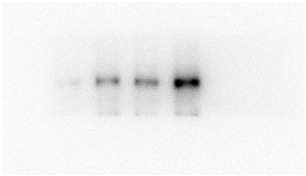

Cleaved-Caspase 3

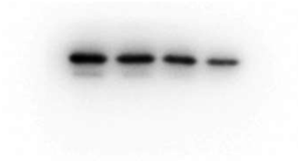

Pro-Caspase 9

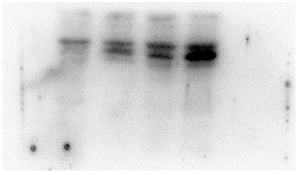

Cleaved-Caspase 9

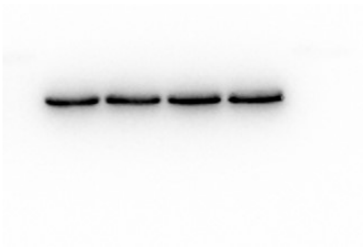

β-Actin

8. Figure 5d

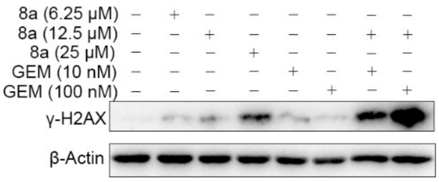

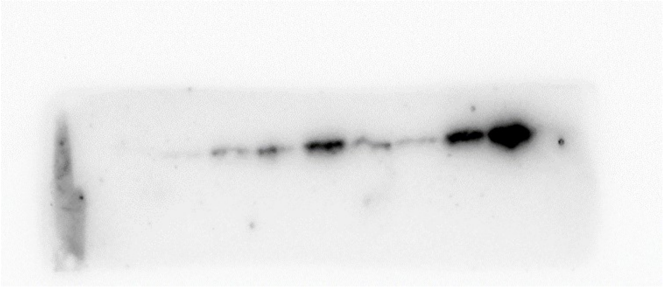

γ-H2AX

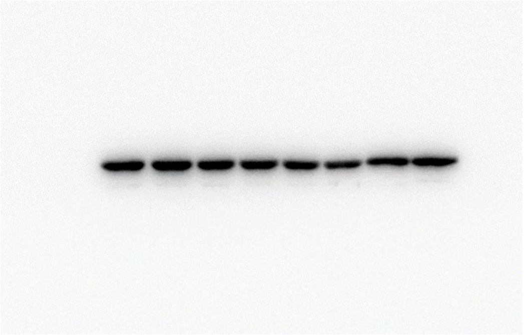

β-Actin

9. Figure S3

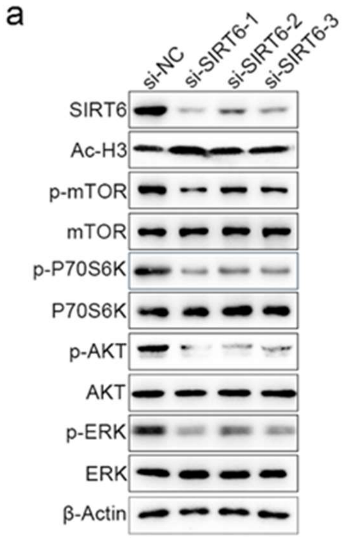
a
si-NC
si-SIRT6-1
si-SIRT6-2
si-SIRT6-3
SIRT6
Ac-H3
p-mTOR
mTOR
p-P70S6K
P70S6K
p-AKT
AKT
p-ERK
ERK
β-Actin

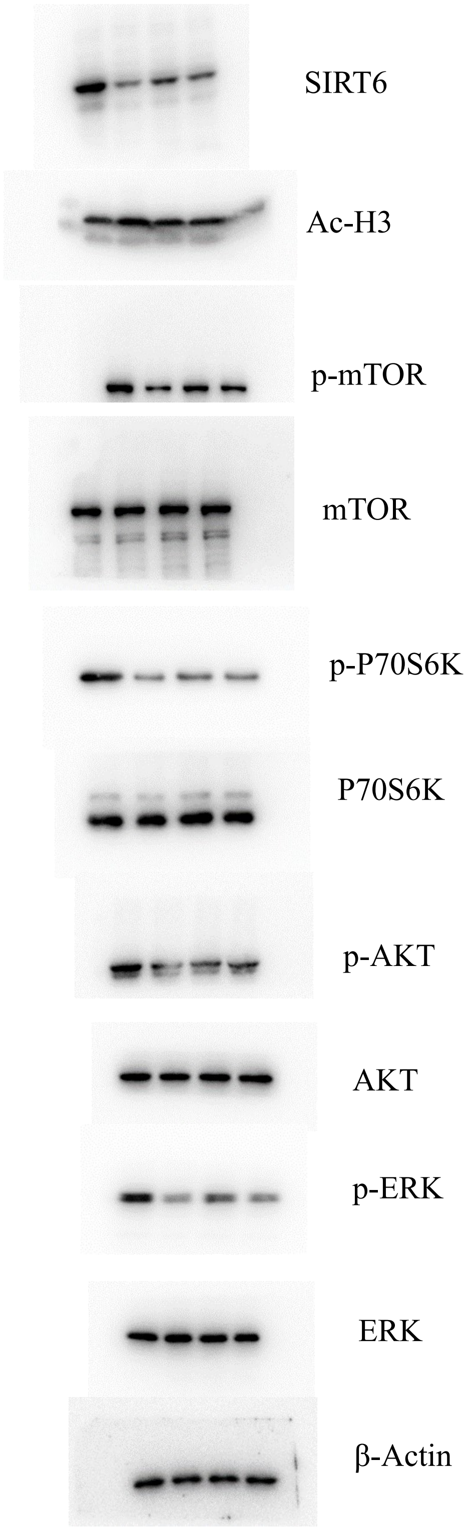
SIRT6
Ac-H3
p-mTOR
mTOR
p-P70S6K
P70S6K
p-AKT
AKT
p-ERK
ERK
β-Actin

10. Figure S4

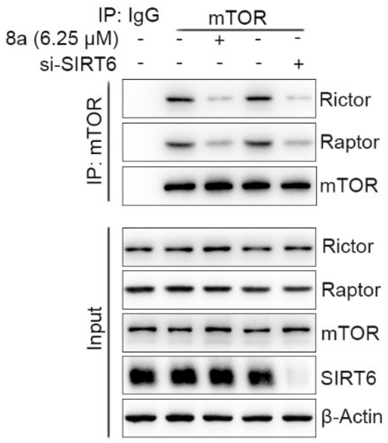
IP: IgG
mTOR
8a (6.25 μM) - - + - -
si-SIRT6 - - - - +
IP: mTOR
Rictor
Raptor
mTOR
Input
Rictor
Raptor
mTOR
SIRT6
β-Actin

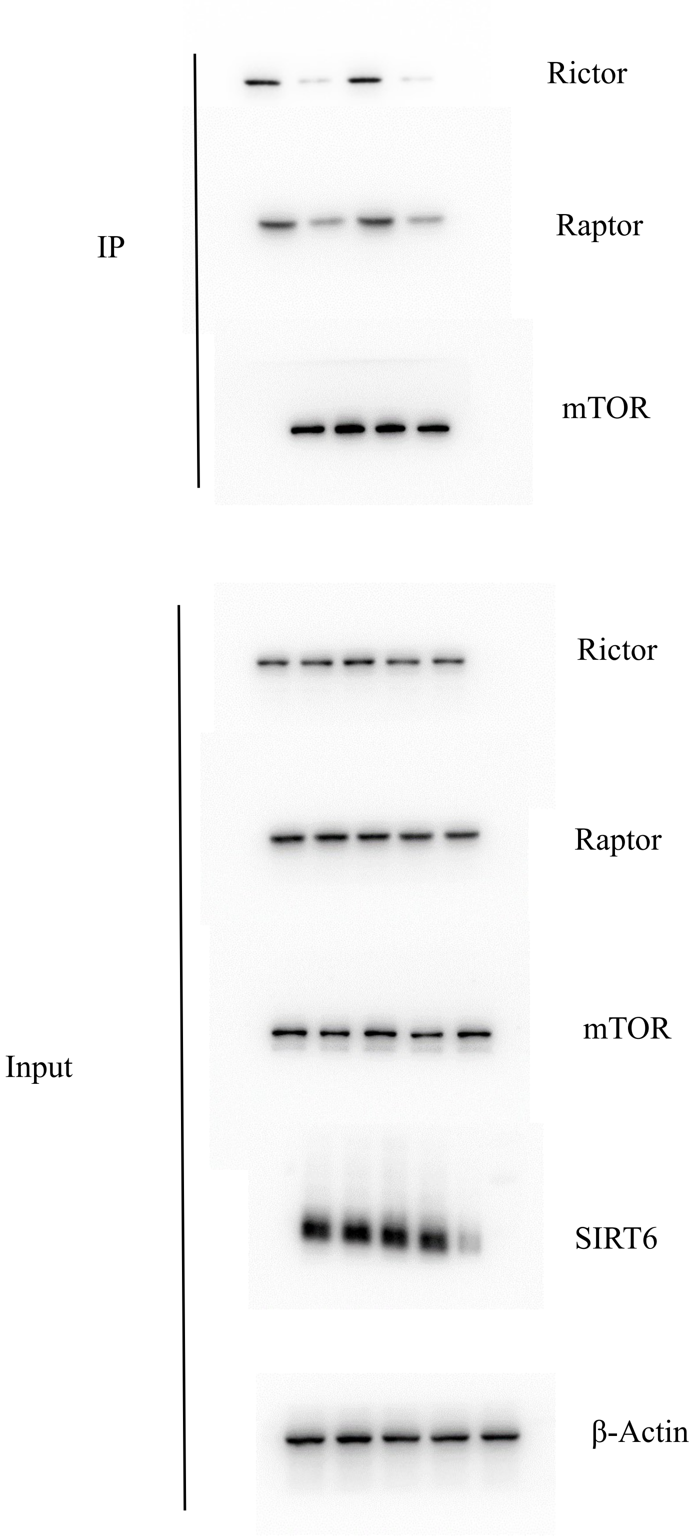
IP
Rictor
Raptor
mTOR
Input
Rictor
Raptor
mTOR
SIRT6
β-Actin

11. Figure S5

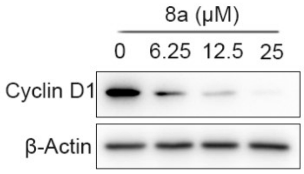

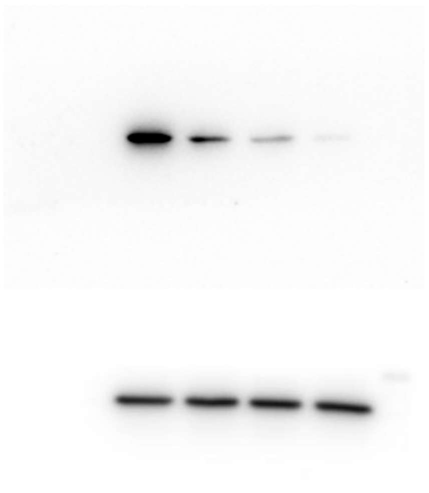

Cyclin D1

β-Actin

12. Figure S6

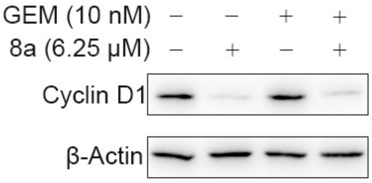

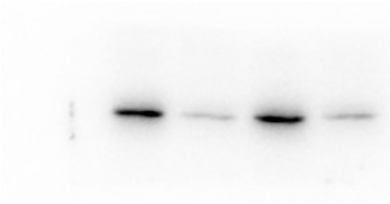

Cyclin D1

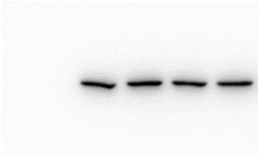

β-Actin

13. Figure S7

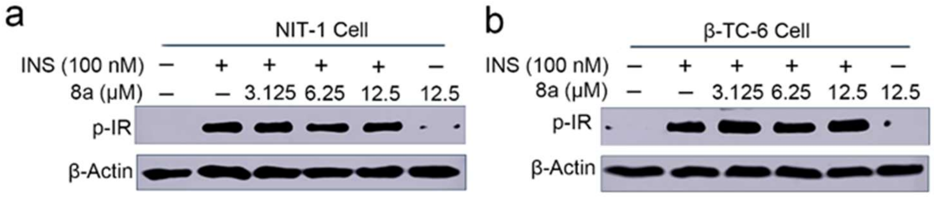

p-IR

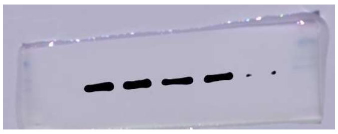

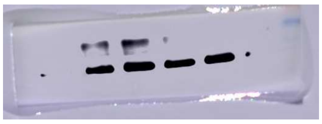

p-IR

β-Actin

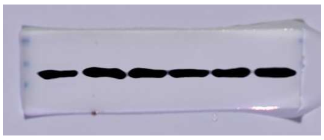

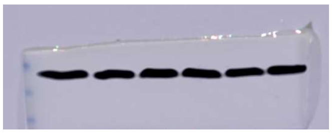

β-Actin
